# Team cognition in remote maritime operations: a scoping review and cross-domain theoretical synthesis

**DOI:** 10.3389/fpsyg.2026.1843838

**Published:** 2026-10-02

**Authors:** Jonas Josten

**Affiliations:** 1 Department of Ocean Space Operations and Civil Engineering, NTNU Norwegian University of Science and Technology, Ålesund, Norway; 2 Norges teknisk-naturvitenskapelige universitet, Ålesund, Norway

**Keywords:** cognitive psychology, distributed cognition, human-autonomy, interactive team cognition, remote maritime operations, situation awareness

## Abstract

**Introduction:**

Remote maritime operations increasingly depend on complex, distributed socio-technical systems in which safe and effective performance relies on team cognition rather than on individual decision-making alone. Despite its importance, the conceptualisation and measurement of team cognition in this domain remain fragmented and underdeveloped.

**Methods:**

A scoping review was conducted following the Preferred Reporting Items for Systematic Reviews and Meta-Analyses extension for Scoping Reviews (PRISMA-ScR). Searches of SCOPUS, Web of Science, and EBSCOhost yielded 162 records, of which 51 publications (35 journal articles and 16 conference proceedings, published between 2010 and 2025) were included after screening, retrieval assessment, and expert recommendation. The included publications were thematically analysed using a hybrid deductive-inductive approach.

**Results:**

Five themes emerged: (1) situation awareness and its measurement and modelling, (2) collective team cognition, learning, and training, (3) human-autonomy teaming and remote collaboration, (4) visualisation and interface design for shared cognition, and (5) maritime applications of distributed cognition. The evidence demonstrates a shift from static, individual-based models toward dynamic, interaction-driven, and system-level accounts, in which team cognition arises from ongoing interactions among human agents, technical artefacts, and environmental constraints.

**Discussion:**

The findings indicate that shared and distributed situation awareness is central to coordination and safety in remote maritime operations. Key challenges include integrating autonomous systems, designing interfaces that support distributed sensemaking, and reliably measuring team cognition. Enhancing remote maritime safety requires aligning design, training, and regulatory frameworks with the principles of distributed cognition. Future research should prioritise longitudinal and intervention-based validation in real-world operational contexts.

## 1 Introduction

Cognition is largely seen as an individual-level variable. It is uniquely individual and often defines you as a person. Thus, thoughts and ideas that could aid others in difficult situations are often empirically measured as distinct entities. When many situations require planning and teamwork, this is somewhat unfortunate, as sharedness of mental models is highly important. This holds true, specifically in multi-operator, complex maritime operations. Furthermore, this is emphasized by real-life maritime incidents.

A much-debated example is the incident of Helge Ingstad and Sola TS in 2018. Here, Helge Ingstad collided with a tanker. One can assume that the team on the bridge had developed a fixed, incorrect mental model, hence falling victim to the confirmation bias (Cummings et al., 2013; Cummings, 2017; Mendel et al., 2011). Team interaction might have become locked in a pattern that further confirmed initial flawed assessments. Due to low cognitive flexibility within the team, they looked for information that confirmed their established perceptions and failed to process disconfirming information (Stanton et al., 2017). Another salient example is the collision between the USS Fitzgerald and the ACX Crystal in 2017. Here, ineffective communication between a bridge team and the Combat Information Centre led to a failure to take action as a give-way vessel. This is a clear example of a breakdown of interactions within a multi-team system.

These incidents illustrate that even with advanced technology, the cognitive and collaborative processes of the human team, alongside their ability to build shared awareness, communicate effectively, and coordinate actions, remain fundamental to maritime safety (Cooke et al., 2007, 2013; Cooke, 2015; Cooke et al., 2024; Demir et al., 2019). What the aforementioned exemplifies is that several cognitive individuals failed to work as one cohesive cognitive system, because their means of sharing their individual thoughts failed, as interactional systems between them did not function optimally. As a result, teams can also fall victim to mental biases often preserved for individual decision makers.

Given the importance of team cognition in complex, multi-operator tasks, this article aims to explore the extent to which team cognition theories are applied in remote maritime operations. An attempt will be made to identify the key aspects of team cognition and how these interact within a broader socio-technical environment. As such, this scoping review is guided by two primary research questions: First, how is team cognition conceptualized and measured in the context of maritime operations? Second, what are the key socio-technical factors that enable or constrain collective awareness and coordination in this domain? By addressing these questions, the review aims to clarify how team cognition emerges from complex interactions of human operators, technical artefacts, and environmental constraints. This is of great interest, seeing how this perspective is increasingly recognized as essential for safe operation and system design (Grand et al., 2016; Stanton et al., 2017). The primary audience for this review is researchers and practitioners in maritime human-factors, cognitive engineering, and human-autonomy teaming. The review adopts a human-systems integration perspective, aiming to synthesize knowledge that can inform the design of team cognitive measures and coordination protocols for remote maritime operation centers.

This scoping review will first define team cognition and its relationship to situation awareness, distributed cognition and human autonomy teaming before presenting the systematic synthesis.

## 2 Individual cognition

We construct our external understanding of the world through internal cognitions (Corbetta et al., 2008; Hutchins, 1995). In maritime operations, this idea is particularly relevant. Much of what operators perceive as situational reality at sea; risk levels, system status, threat detection, or urgency, emerges from how they interpret and mentally construct incoming information (Endsley, 1995).

For example, an operator may interpret an ambiguous radar signal as an immediate threat, whereas another may treat the same signal as manageable uncertainty (Beck, 1991; Wickens, 2002).

Because maritime professionals operate within bridge teams and remote operation centers, individual interpretations must be coordinated with colleagues, procedures, technical systems and changing environmental conditions (Cooke et al., 2007; Lacerenza et al., 2018). These differences may undermine coordination, but they may also introduce alternative viewpoints and reduce cognitive bias when they are openly discussed (DeChurch and Mesmer-Magnus, 2010; Lacerenza et al., 2018; Rand, 2016).

Our individual cognition, however, must work in tandem with others’ cognition. In the larger society, and in cooperative operations, this is especially relevant.

### 2.1 Social cognition

Humans are inherently social decision-makers (Lee and Harris, 2013). Effective maritime performance depends not only on accurate individual cognition but also on the ability to integrate multiple perspectives, reconcile conflicting interpretations, and construct a shared operational picture (Fiore et al., 2003; Hutchins, 1995). In this sense, safe and effective maritime operations require mastery of both internal cognitive processes and external social coordination. Operators must manage their own interpretations while remaining open to revision through dialogue, feedback, and emerging evidence, especially in dynamic, high-consequence maritime environments (DeChurch and Mesmer-Magnus, 2010).

Social cognition is highly relevant to maritime operations because team members must integrate perspectives (Fiore et al., 2003; Hutchins, 1995; Straus et al., 2011), communicate emerging information (Cooke, 2015; Cooke et al., 2007, 2013, 2024), and revise interpretations under changing conditions (DeChurch and Mesmer-Magnus, 2010). Social facilitation, reduced effort, conformity, and suppression of dissent may affect team cognition when they restrict communication or critical challenge, but these processes are treated as team-process conditions rather than as independent theoretical foundations. As such, this review focuses on team cognition as an interactional and socio-technical process rather than on general group psychology (i.e., Bénabou, 2013; Cottrell et al., 1968; Lange and Washburn, 2012; Lott and Lott, 1965; Rockloff and Dyer, 2007; Tutlani and Kumar, 2024).

These dynamics highlight that cognition in groups is not merely the sum of individual thoughts, but shared interactive processes (Cooke, 2015; Cooke et al., 2007, 2013, 2024). This perspective leads directly to the concept of team cognition, which examines how knowledge, mental models, and information processing are distributed and coordinated across members to enable or constrain effective collective performance.

#### 2.1.1 Team cognition: shared, interactive and distributed perspectives

The previous paragraph alludes to team cognition, and cognitions spread across several actors towards a common goal. This idea of distributed cognition becomes exceedingly important in high-risk, highly complex environments such as remote maritime operations. These environments are complex socio-technical networks in which humans must cooperate with other humans and technology simultaneously (Cooke et al., 2024; Demir et al., 2019; Kari and Steinert, 2021; Lim et al., 2018; Strauch, 2018).

Multi-operator tasks require complex individual and social cognitive processing (Cooke et al., 2000). As previously discussed, many complex cognitive processes occur at the individual level; however, research suggests that teams function as cognitive units (Gorman, 2014; Gorman et al., 2020; Van Eijndhoven et al., 2023). As such, teams can become more than the sum of their parts. Generally, team cognition is reliant on team mental models, defined as a shared understanding of tasks, goals, and environments, transactive memory systems that emphasize knowledge distribution and collaboration, and team situational awareness, which is a shared understanding of current and future situations and events (Cooke et al., 2013; DeChurch and Mesmer-Magnus, 2010; Esmaeili et al., 2025). In short, team cognition can be defined as emergent collective cognition (DeChurch and Mesmer-Magnus, 2010).

However, team cognition is multifaceted. As such, one must also understand how specific, relevant and useful cognitions are shared, not just what they are. Accordingly, there will now be a discussion of sharedness and interaction.

DeChurch and Mesmer-Magus (2010) state that team cognition is an emergent state that encompasses team-important knowledge sharing, the sharedness of mentally organized knowledge, and its distribution across team members, enabling the team to predict outcomes more effectively and execute actions. Shared cognition emphasizes the organization and compatibility of knowledge across team members, whereas interactive cognition emphasizes how knowledge is exchanged and coordinated during task performance (Cooke et al., 2013; Cooke, 2015).

This assumption of similarity as beneficial implies that shared cognition thrives in environments where team members think alike, potentially promoting efficiency and reducing misunderstandings. However, it is important to question this assumption, as heterogeneity within a team might foster diverse perspectives and spur innovative solutions. Reflecting on when and how diversity in team composition might be advantageous can broaden the understanding of effective team contexts.

Cognition can be viewed as something that should be measured at team level, not only at the individual level (Cooke et al., 2013). This is the view of Interactive Team Cognition [ITC]. While shared cognition focuses on accumulated knowledge between agents, interactive cognition would emphasize how individual information and knowledge are shared through interaction (Cooke et al., 2000, 2013; Cooke, 2015; Cooke et al., 2024; Demir et al., 2019). As a result, this theory focuses on cognitive processes rather than cognitive structures. The process in question is the interaction between team members (Cooke et al., 2013). ITC posits that team interactions aggregate shared knowledge as needed, rendering knowledge aggregation and the emergent team knowledge state redundant. For example, successful information handoffs serve as a measurable cue of healthy ITC. For example, Cooke and Gorman (2009), introduced interaction-based metrics that quantified mission-critical information, successfully passed from one teammate to another. These information handoff rates correlate strongly with both real-time situational awareness, and final mission success, providing an observable cue for healthy, shared and interacted cognitions among operators (Cooke et al., 2013).

To concretize, this review will treat cognitive interaction as the exchange or transformation of task-relevant information that changes, tests or coordinates teams evolving representation of the task, goal, plan or operational situation. Moreover, communication frequency, network centrality, information handoffs, and coordination behaviors are treated as behavioral or relational indicators (Cooke and Gorman, 2009; Cooke et al., 2013). As such, they are interpreted as evidence related to team cognition only when the available data show that the interaction carries task-relevant content, is taken up for transformation by another actor, and contributes to a change in shared understanding, planning coordination, or situation awareness. Communication frequency alone is therefore not treated as evidence of team cognitive processes.

Herein lie the sentiments of situation awareness, a crucial factor for safe and successful multi-operator tasks in dynamic, complex environments such as remote maritime operations. As with other individual psychological factors, situation awareness must also be shared between several individuals. The following section will outline how this might occur.

## 3 Situational awareness

Due to the dynamic nature of complex environments and human-system interactions, operators are expected to have an accurate understanding of both developing situations and current states (Endsley, 2000). Due to the demand placed on operators, situational awareness is a key factor for ensuring safe and efficient operations in most task-demanding occupations (Endsley, 1997; Niessen et al., 1999). Endsley (2000) promotes three levels of situation awareness: mainly perception of critical data/stimuli, comprehension of said critical data/stimuli, and projection, meaning predictions of future actions and situations. Usually, situational awareness requires an individual’s mental model of the previously discussed concepts. These models often include dynamic task properties such as future spatial and temporal relationships, as well as unexpected complications (Wickens, 2002). In dynamic, complex settings, maintaining accurate situational awareness requires an individual’s ability to continuously update their understanding of current and future events (Endsley, 1995, 2015, 2017).

### 3.1 Distributed cognition (situation awareness as a distributed concept)

Multi-operator tasks require situation awareness to be shared and distributed among several individuals (Fiore et al., 2003; Hutchins, 1995). This requires a shared understanding of goals, risks, knowledge, skills, and their fellow operators. Additionally, it requires an approximate understanding towards a given situation (Artman and Garbis, 1998.; Fiore et al., 2003). Furthermore, by distinguishing between sharedness and distribution, situation awareness receives a more dynamic meaning. To share situation awareness, one usually implies that any member shares a degree of overlap or commonality in the concept (Perla et al., 2000). Sharedness is often viewed as a prerequisite for effective coordination due to the notion that collected components of individual situation awareness should be held in common (Javed et al., 2011). However, team members do not need identical knowledge. In fact, they should show compatible awareness that allows for successful coordination (Stanton et al., 2006). Viewing situation awareness as a distribution gives a more dynamic and nuanced view of its importance. For instance, team situation awareness is not simply transferred between humans (Artman and Garbis, 1998). However, it is actively constructed through communication (Artman and Garbis, 1998.; Cooke, 2015; Demir et al., 2019). This entails negotiating solutions and compromises, sense-making, and the development of a shared consensus (Perla et al., 2000). In this way, individuals can integrate each other’s mental models to create a shared or compatible understanding (Jones et al., 2021; Perla et al., 2000).

Operating remotely controlled maritime vessels requires navigating a highly technical and complex environment. Since no individual is a passive agent to their surroundings but constantly makes sense of everything around them, mistakes occur (Kahneman, 2003, 2012). This results from intense cognitive pressure and the brain’s tendency to form cognitive shortcuts to compete in society (Hilbert, 2012; Cummings, 2017; Eberhardt, 2019; Tversky and Kahneman, 1973). As environments become increasingly technological, humans must work alongside machines, technical objects, and other humans (Cooke et al., 2024; Demir et al., 2019a; Kari and Steinert, 2021; Lim et al., 2018; Strauch, 2018). As such, tools and technologies become active components of our cognitive systems. (Fiore et al., 2003). These tools and technologies can function as cognitive artefacts that aid, enhance or externalize cognitions (Fiore et al., 2003). To understand this, one must discuss human-autonomy pairings.

## 4 Human-autonomy pairing

Interactions are no longer preserved only for humans. More often, humans now face artificial and autonomous technological agents they need to cooperate with. Cummings (2017) reports on biases specific to automated environments. These take the form of automation, anchoring, and confirmation bias. What these entail are the over- or under-reliance on automated programs, the tendency to rely too heavily on the first piece of information offered, and the drawing towards information that supports our assumptions (Cummings et al., 2016; Hutton and Klein, 1999; Kahneman, 2012). As a result, operators are unable to adapt to complex situations due to mental inflexibility in decision-making (Eberhardt, 2019; Tversky and Kahneman, 1973). This emphasis is critical because when operators fall prey to such biases, it often results in a significant degradation of situational awareness (SA), as they become complacent and process less of the raw information directly related to the automated tasks (Chen et al., 2017; Cummings, 2017; Cummings et al., 2013, 2016).

Automated environments often rely on human-in-the-loop approaches to keep human operators aligned with autonomous agents (Cummings et al., 2013, 2016; Mosqueira-Rey et al., 2023; Pattyn et al., 2008; Veitch and Andreas Alsos, 2024). Such an approach is designed to reduce human error and workload. However, this approach can complicate the operations of unmanned vessels when humans fall out of the loop. Complications as omission, that is, failure to detect problems due to the automation not alerting properly, or/and commission, erroneously following automated directives might arise (Cummings, 2017; Cummings et al., 2013, 2016). Such automation-induced complacency not only compromises the operator’s understanding of their task environment but can also lead to skill degradation over time, making it difficult to regain manual control effectively during infrequent or unpredictable interventions. Conclusively, this underscores the importance of keeping the operator within the operations, locked into their cognitive environment, and designing systems with the cognitive limitations of humans in mind (Cummings et al., 2013, 2016). Human autonomy teaming is therefore treated as an operational boundary condition that shapes information exchange, situation awareness and coordination, within distributed teams. Its specific implications for remote maritime operations are examined in the thematic analysis.

The reviewed literature uses several related constructs to describe collective cognition. However, these constructs operate at different analytical levels. In this review, team cognition is treated as an umbrella construct. Shared or team mental models and transactive memory systems describe the organization of knowledge. Furthermore, interactive team cognition describes cognition enacted through interaction. Moreover, team situation awareness describes situation specific collective awareness. Additionally, distributed cognition provides a broader socio-technical perspective on how cognitive work is distributed across people artefacts, technologies and the environment (Table 1).

**Table 1:** Conceptual Distinctions among Central Team Cognitive Constructs.

Construct	Primary status	Unit or level of analysis	Core distinction
Team cognition	Umbrella construct spanning cognitive structures, cognitive processes, and emergent team-level properties.	Team, multiteam system, or socio-technical system.	Encompasses the organization, exchange, transformation, and use of task-relevant knowledge. It is not equivalent to any single component (Cooke et al., 2013; Cooke, 2015; DeChurch and Mesmer-Magnus, 2010).
Shared or team mental models	Cognitive structure, with team-level emergence when member representations become compatible or complementary.	Individual representations considered in relation to the team or dyad.	Concerns the content, similarity, compatibility, accuracy, or quality of task, role, goal, and environmental representations. It does not by itself demonstrate the interaction through which knowledge is developed or enacted (Mathieu et al., 2000; Mohammed et al., 2010; Resick et al., 2010).
Transactive memory system	Distributed cognitive structure plus a retrieval and coordination process.	Team or knowledge network, with expertise distributed across individuals.	Concerns specialized expertise and knowledge of who knows what. Members need not possess identical knowledge; the shared element concerns access to distributed expertise (Shteynberg et al., 2020).
Team situation awareness	Emergent team state maintained through an ongoing cognitive process.	Team, operational unit, or distributed system.	Concerns perception, comprehension, and projection of the current or anticipated operational situation. It is a situation-specific manifestation of team cognition, not a synonym for team cognition as a whole (Endsley, 1995; Gorman et al., 2006).
Interactive team cognition	Cognitive process enacted through team interaction.	Dyad, team, or interaction network.	Concerns task-relevant information exchange, interpretation, transformation, anticipation, and coordination during task performance. Interaction is not automatically cognition; task content and cognitive uptake must be identifiable (Cooke et al., 2013; Cooke, 2015; Cooke and Gorman, 2009).
Distributed cognition	Socio-technical theoretical perspective and system-level explanatory framework.	Distributed cognitive system, including people, artefacts, technologies, and environment.	Explains how cognitive work is distributed across the wider system. It is broader than team cognition and does not require a bounded team or a shared mental representation (Hutchins, 1995; Stanton, 2014).
Communication and coordination	Behavioral or relational process, and possible mechanism or indicator of cognition.	Dyad, team, network, or system.	Observable activity through which team members exchange information, align activity, manage dependencies, and perform handoffs. These behaviors are not cognitive states in themselves (Cooke and Gorman, 2009; Roberts et al., 2019).
Interfaces, cognitive artefacts, and operational environment	Technological or environmental affordance and contextual condition.	Human-system interface, control room, vessel, network, or wider socio-technical system.	Conditions that support or constrain cognition by shaping information visibility, access, integration, and distribution. They are not themselves team cognition, a mental model, or team situation awareness (Hutchins, 1995; Javed et al., 2011; Song et al., 2023).

The categories are analytically distinct but may be empirically related. Communication and coordination are treated as observable behavior or relational processes, whereas. Interfaces, artefacts and operational environments are treated as contextual conditions or affordances.

The following section will discuss methods for selecting and analyzing the literature synthesized in this review.

## 5 Methods

This study was designed as a scoping review to map how team cognition has been conceptualized, measured and applied across remote maritime and comparable safety-critical domains. Reporting followed the Preferred Reporting Items for Systematic Reviews and Meta-Analyses extension for Scoping Reviews (PRISMA-ScR; Tricco et al., 2018). The review was not prospectively registered on a public registry. The initial database search across SCOPUS, Web of Science and EBSCOhost yielded 162 records. After removal of duplicates (*n* = 22), 140 records were screened. In total 75 records were excluded before retrieval, comprising of 43 exclusions based on title screening, 8 review articles, and 24 exclusions based on abstracts. Sixty-five reports were sought for retrieval. Sixteen could not be retrieved, leaving 49 reports to be assessed for eligibility from database searches. Further 2 publications were included based on expert recommendation, resulting in a final corpus of 51 articles included in the synthesis. The complete PRISMA-ScR flow diagram is presented in Figure 1.

**Figure 1:**
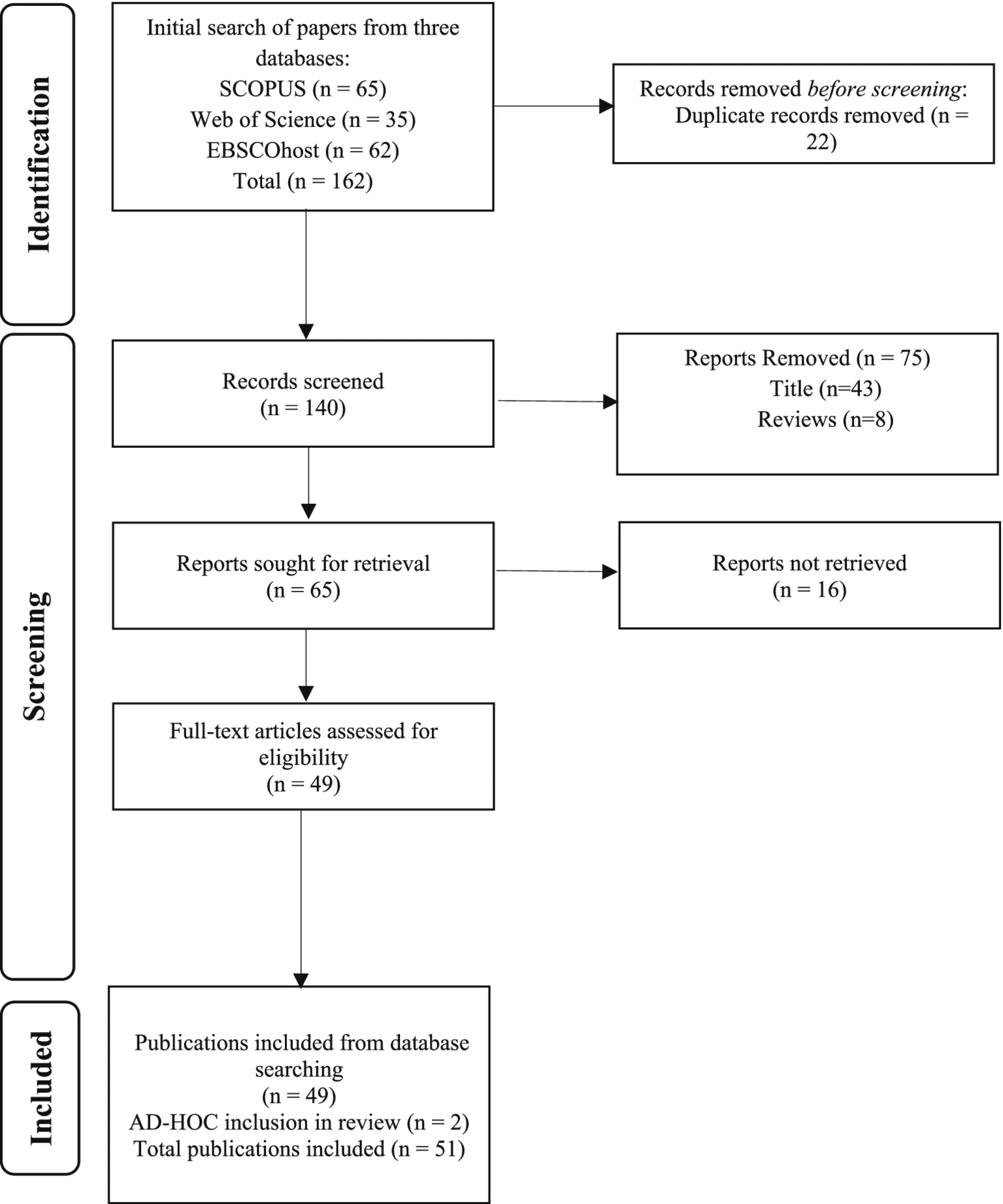
PRISMA flow diagram of the review process. Number of documents identified, inclusions, and exclusions.

Title, abstract and full-text screening were conducted by the first author using the predefined eligibility criteria. To improve consistency, the screening decision was checked against an eligibility framework, and ambiguous cases where discussed with peers. Disagreements or uncertainty were resolved through discussion and documented in the screening file. Because screening was not conducted independently by two reviewers, no inter-rater reliability statistic is reported. This limitation is acknowledged in the discussion.

The thematic analysis followed a hybrid deductive-inductive approach. The initial coding framework was informed by established theories of team cognition. Specifically, distributed cognition (Hutchins, 1995; Stanton et al., 2006), and interactive team cognition (Cooke, 2015). These provide the deductive anchor for this thematic analysis. Furthermore, open coding was applied to the full corpus of 51 articles in order to capture initial, emergent constructs. Iteratively, codes were refined through discussions with peers, merged where overlapping, and grouped into candidate themes. Further theme development followed the six-phase reflexive thematic analysis proposed by Braun and Clarke (2006). The five final themes were stabilized after several rounds of review against the coded data. Regarding inclusion of ambiguous cases, such as articles addressing team performance without explicitly invoking any notions of cognition, were retained only if a logical link to one or more of the five thematic constructs could be established.

Moreover, the thematic analysis followed six sequential phases. First, the author familiarized themselves with the included publications by reading the full texts and recording initial observations concerning constructs, measures, operational contexts, and reported outcomes. Second, initial codes were generated using a hybrid deductive inductive strategy. The deductive codes informed by distributed cognition and interactive team cognition while, open coding captured constructs that emerged from the publications. Third, related codes were grouped into candidate themes. Fourth, candidate themes were reviewed against the coded extracts and the full set of included publications, with overlapping or insufficiently distinct codes merged or revised. Fifth, each final theme was defined according to its central construct, unit of analysis, operational context, and relationship to team cognition. Sixth, the final themes were synthesized comparatively with attention to methodological strength, direct maritime relevance, cross-domain transferability and conceptual boundaries.

Coding was conducted by the author. Ambiguous coding decisions were resolved through peer discussion. The final codebook, including code definitions, cross domain transferability and conceptual boundaries are provided in the Appendix Table A2–A6.

### 5.1 Defining the scope and keywords

The following steps were taken to systematically review the discussed topics.

First and foremost, the area of team interaction within remote operation became of interest. As such, relevant databases were selected following the procedures of Webster and Watson (2002). The digital libraries identified as most relevant were SCOPUS, Web of Science, and EBSCOhost. It is acknowledged that other databases, such as IEEE Xplore, Taylor & Francis, or ScienceDirect, may be relevant. However, these were excluded as the literature featured in them is expected to be included in the chosen databases. Lastly, to ensure a specific and up-to-date search of relevant literature, restrictions were applied to publication year and field of interest. The publication year range from 2010 to 2025, and the scientific field is remote maritime operations.

Care was also taken in constructing the search strings for each database. Measures were taken to ensure conceptual similarity while still leveraging the strengths of each database. Key terms such as team cognition (Cooke, 2015; Cooke et al., 2007, 2013; Grand et al., 2016), and remote operations were ensured in all databases. However, each search string varies slightly across databases. Searches were conducted in SCOPUS, Web of Science, and EBSCOhost to ensure both specificity and comprehensive coverage across engineering, maritime, and psychological literature. Database-specific syntax and indexing practices were respected, and search strings were adapted accordingly. Results were exported and merged, with duplicates removed prior to screening. Table 2 provides an overview of the search strings.

**Table 2:** Search strategy table

Database	Platform	Search Fields	Search String	Limits	Retrieved
SCOPUS	Elsevier SCOPUS Version 2025 Searched 16th of December 2025	TITLE-ABS-KEY	(TITLE-ABS-KEY (“remote operation*” OR “remote control” OR “remote operating centre*” OR “control room*” OR “supervisory control” OR “autonomous vessel*” OR “maritime autonomy” OR “remote ship” OR “remote navigation” OR “uncrewed vessel*” OR “USV” OR “MASS”)) AND (TITLE-ABS-KEY (“interactive team cognition” OR “team cognition” OR “shared cognition” OR “transactive memory” OR “distributed cognition” OR “distributed situational awareness” OR “team situation* awareness” OR “team SA” OR “shared situation* awareness”)) AND PUBYEAR > 2009 AND PUBYEAR < 2026	English; Document type: Article, Conference paper. Publication year: 2010 to 2025.	65 documents
Web of Science	Clarivate WoS Core Collection Version 2025 Searched 16^th^ of December 2025	TS(Topic = TI, AB, AK, KP)	TS = ((“remote operation*” OR “remote operating centre*” OR “control room*” OR “supervisory control” OR “autonomous vessel*” OR “remote vessel” OR “maritime autonomous surface ship*” OR MASS OR USV) AND (“interactive team cognition” OR “team cognition” OR “shared cognition” OR “distributed cognition” OR “shared situation* awareness» OR “distributed situation* awareness” OR “transactive memory”))	English; Document type: Article, Conference paper Publication year: 2010 to 2025	35 documents
EBSCOhost (e.g., PsycINFO, Academic Search Complete, APA)	EBSCOhost Version 2025 Searched 16^th^ of December 2025	TI, AB, SU, DE	(TI(“remote operation*” OR “remote operating centre*” OR “remote control room*” OR “supervisory control” OR “remote monitoring” OR “autonomous vessel*” OR “autonomous ship*” OR “uncrewed vessel*” OR USV OR MASS OR “maritime autonomy” OR “remote navigation”)) AND ((“interactive team cognition” OR “team cognition” OR “shared cognition” OR “shared mental model*” OR “transactive memory” OR “distributed cognition” OR “team situational awareness” OR “shared situational awareness” OR “distributed situational awareness”)) AND ((“human factor*” OR “human-in-the-loop” OR “human automation interaction” OR “human-machine interaction” OR “cognitive load” OR “mental workload” OR “decision-making” OR vigilance OR attention OR sensemaking OR heuristic* OR bias*))	English; Document type: Article, Conference paper Publication year: 2010 to 2025	62 documents

Conceptually similar and database-specific search strings. Limitations are ascribed to the database and documents identified. All three searches were run on the same day of 16th of December 2025.

The current review targets the era of remote maritime operations and maritime autonomy (MASS, remote control centers, autonomous vessels), which accelerated from 2010 onward, coinciding with the emergence of the relevant operational literature (e.g., Kari and Steinert, 2021; Lynch et al., 2023). For methodological reasons, a scoping review of contemporary literature requires a bounded time window to keep the corpus manageable and current. However, as such there occurs a risk of omission. Mitigation of omission risk is done by not excluding foundational pre-2010 work such as Hutchins (1995), Endsley (1995), Cooke et al. (2000), Stanton et al. (2006), and DeChurch and Mesmer-Magnus (2010). On contrast they are used as theoretical anchors, even though the reviewed corpus is restricted from 2010 to 2025.

The search strategies were conceptually aligned across SCOPUS, Web of Science, and EBSCOhost. However, they were not syntactically identical. Each database search contained two common conceptual blocks: one addressing remote maritime operations, and one addressing team cognition and related constructs. The strategy for EBSCOhost included an additional block addressing human-factors, human-in-the-loop systems, cognitive load, workload, decision-making, vigilance, attention, sensemaking heuristics and cognitive bias. This addition was introduced to account for the indexing structure and controlled vocabulary of the EBSCOhost collections used at the time of the original search. It was intended to improve conceptual retrieval within the EBSCOhost environment rather than introduce an additional eligibility criterion.

Furthermore, the conceptual correspondence among the database strategies therefore defined at the level of the review concepts rather than through identical syntax. The first block represented the operational context, the second represents team cognition and related cognitive structures, and the additional EBSCOhost block represents related human-factors and cognitive mechanisms. Database-specific field structures and indexing practices required adaptations to the Boolean syntax and searchable fields.

Due to the EBSCOhost platform and database configuration changed after the original search an exact reproduction of the historical search was not possible. The original search was conducted 16th of December 2025, and in August 2026, the platform rolled out a major new interface update that changed default search modes, folder structures and how queries and limits were processed. Therefore, a functional audit was conducted using the closest available current platform configuration. The reconstructed two block EBSCOhost search returned 98 records, while the corresponding three-block search returned 86 records. The additional conjunctive block therefore excluded 12 records, representing 12.2% of the two-block result set under the current platform configuration. These current exports were drawn from different database configurations and did not reproduce the historical record set. As a result, the audit is therefore interpreted as a platform-dependent sensitivity assessment rather than an exact replication of the original study.

The main concepts up for review were “team cognition” and “remote maritime operations”. Related search terms like “transactive memory” (Esmaeili et al., 2025; Sun et al., 2022), “distributed cognition” (Artman and Garbis, 1998; Button, 2008; Fincannon et al., 2011), and “distributed situation awareness” (Song et al., 2023; Stanton et al., 2006), “shared situation awareness” (Adamu et al., 2015; Jones et al., 2021; Kim et al., 2019; Perla et al., 2000; Schei and Giske, 2020; Sorsa, 2020; Törmä et al., 2025) and “team situation awareness” (Atweh and Riggs, 2025; Coolen et al., 2019; Crozier et al., 2015; Demir et al., 2019; Grimm et al., 2018; Schei and Giske, 2020; Sorsa, 2020) were also up for consideration. Moreover, there were terms such as “remote operation centers” (Kari and Steinert, 2021), “control room,” “uncrewed vessel” (Cummings et al., 2013; Fincannon et al., 2011; Lim et al., 2018; Lynch et al., 2023), “USV,” and “MASS” included. This would ensure conceptual similarity, nuance, and specificity.

To further ensure conceptual equivalence, another block of search words was added to EBSCOhost. This was to compensate for its indexing differences (PsycINFO + Academic Search Complete) as they have a different controlled-vocabulary. Conceptually, this block is present within all three databases even though there is a difference in syntax. In short, where syntax differs, the block was adapted to the platforms field structure while preserving conceptual content.

The analyzed literature spanned from 2010 to 2025 (*M* = 3.2 per year). Figure 2 presents a graphical representation of the publication year distribution of the 51 included articles. Moreover, there is a peak of publications in 2020 (*n* = 5) and 2021 (*n* = 4), which is indicative of increased research attention on maritime autonomy and remote operations during a period of accelerated digitalization in the maritime sector (Kari and Steinert, 2021; Lynch et al., 2023).

**Figure 2:**
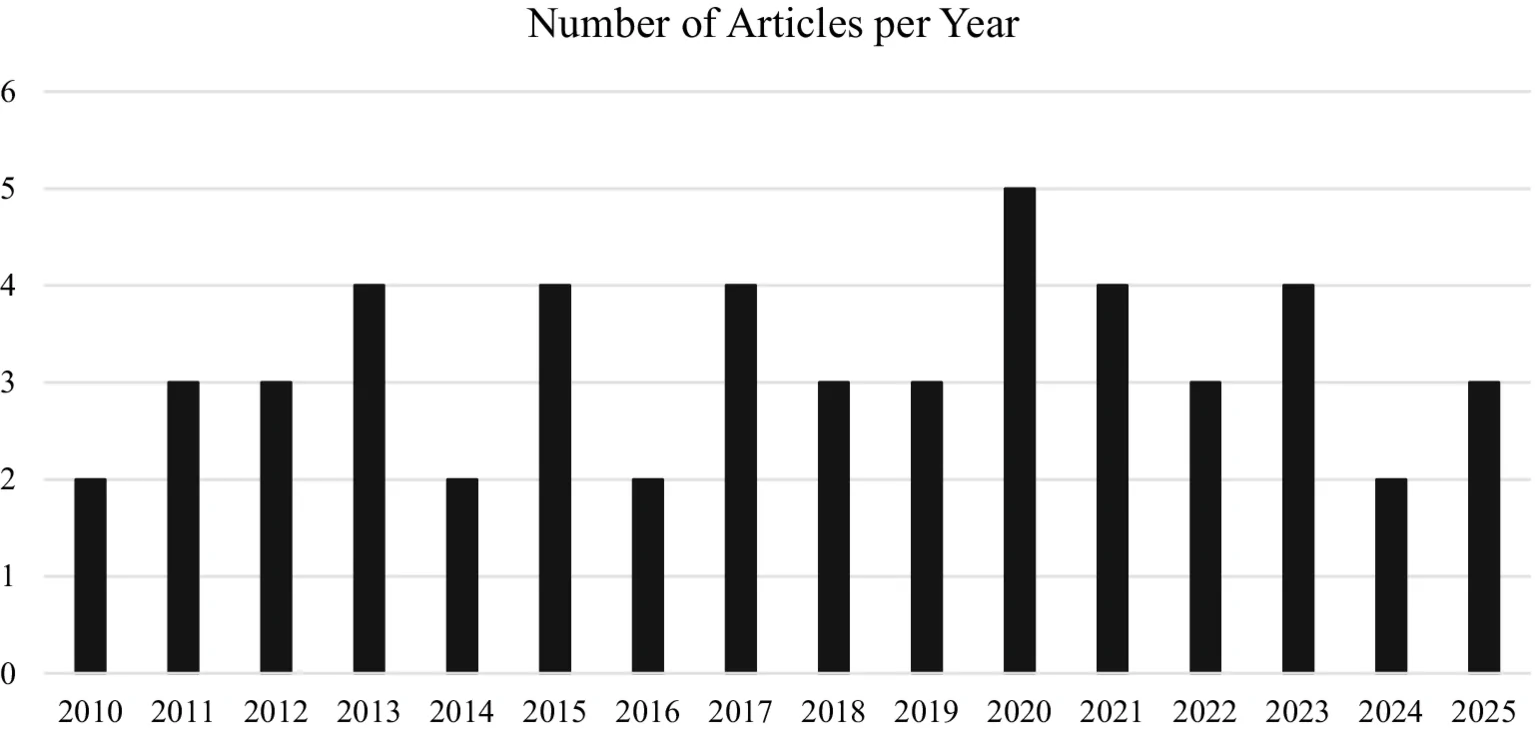
Publication year distribution of included studies (*n* = 51). Graphical representation of publication trends within the analyzed corpus.

### 5.2 Initial search in three digital databases

For the database searches in SCOPUS, Web of Science, and EBSCOhost, the related search strings are listed in Table 2. Furthermore, the results included peer-reviewed scientific journal articles across domains such as engineering, maritime, and psychology, as well as relevant conference papers. They were written in English and published between 2010 and 2025. Collectively, the initial search yielded 162 articles. As such, they were ready for screening. Table 3 presents the inclusion and exclusion criteria for this review.

**Table 3:** Inclusion and exclusion criteria for further analysis.

Criterion	Inclusion	Exclusion
Population	Teams/operators in safety-critical domains	Single-operator studies without team dimension
Concept	Team cognition, SA, coordination, HAT	General automation without cognitive focus
Context	Remote or distributed maritime operations (including analogues: nuclear, aviation, submarine, emergency management)	Non-safety-critical domains; purely technical (hardware/software only)
Source type	Peer-reviewed journal articles, conference proceedings	Grey literature, editorials, opinion pieces

Inclusion and exclusion criteria for further analysis.

### 5.3 Removing duplicates

All articles were entered into an Excel spreadsheet, initially categorized by the database from which each article was obtained. Later, they were sorted by title to facilitate the removal of duplicates. After removing duplicates (*n* = 22), the review was left with 140 articles.

### 5.4 Filtering following exclusion criteria

After removing the duplicates (*n* = 140), the remaining articles were excluded based on their titles, review articles, and abstracts. Articles whose titles did not relate to any of the core concepts for this review were excluded (*n* = 43). These titles were irrelevant to remote maritime operations, team cognition, or any subsequent concepts related to them. By excluding titles (*n* = 43) in which the context of team interaction was not relevant, nor in the scientific field in question, this review was left with 97 articles.

Of the remaining articles (*n* = 97), eight were identified as reviews that had not been filtered *a priori*. As such, leaving (*n* = 89) to be scrutinized through their abstracts.

A further 24 articles were excluded based on their abstracts. As a result, 65 articles remained. Furthermore, when attempting to access the full-text articles, 16 were unobtainable because the author did not receive responses from the owners of the specific articles, resulting in 49 articles being assessed. *Ad hoc*, 2 relevant articles were recognized and included. Meaning, this review includes (*n* = 51) full-text articles.

### 5.5 Evidence characterization and registration

Because this review was designed to map heterogenous evidence base rather than estimate a pooled intervention effect, no single conventional risk of bias instrument was applied across all included publications. Instead, each publication was characterized using a structured evidence appraisal framework covering five dimensions: (C1) purpose and design transparency, (C2) context and evidence source transparency, (C3) construct and measurement transparency, (C4) analysis or theoretical-derivation transparency, and (C5) directness and transferability to remote maritime operations. Each dimension was coded as clearly reported, partially reported, not reported or not applicable, depending on the publications purpose and design. The appraisal was conducted by the first author, with ambiguous coding decisions discussed with peers. It was useful to qualify the interpretation of the synthesis rather than to exclude publications retrospectively. The evidence-characteristics framework was developed from the review objectives and refined iteratively during initial coding. It was applied to all 51 included publications. Findings based on direct maritime evidence and clearly reported empirical methods were distinguished from findings based primarily on conceptual argument, cross domain analogy or limited reporting. The short evidence-characterization matrix is provided in Table 4.

**Table 4:** Operational theme-to-criterion crosswalk.

**Theme code**	**Primary criteria**	**Reason for emphasis**
SA/M	C2, C3, C4, C5	Context, measurement, analytical modelling, and transferability are central to situation-awareness studies.
CTC/L/T	C1, C3, C4, C5	These studies require attention to purpose, construct operationalisation, theoretical or analytical derivation, and operational transferability.
HAT/RC	C1, C2, C3, C5	Human-autonomy and remote-collaboration studies require scrutiny of design, operational context, measures, and transferability.
V/ID	C2, C3, C5	Interface studies require scrutiny of context, visualisation or measurement procedures, and relevance to remote operations.
M/DC	C2, C4, C5	Distributed and maritime cognition studies require scrutiny of system context, analytical derivation, and maritime transferability.

Appraisal criteria: C1 = purpose and design transparency; C2 = context and evidence-source transparency; C3 = construct and measurement transparency; C4 = analysis or theoretical-derivation transparency; C5 = directness and transferability to remote maritime operations. Coding rule for the completed evidence-characterization appraisal. C: Clearly reported. P: Partially reported. N: Not reported. NA: Not applicable to the publication type or study purpose.

The corpus included experimental, observational, conceptual, methodological and conference publications for which different appraisal criteria are appropriate. As such, each included publication was characterized using a structured appraisal framework covering five dimensions: clarity of the study aims, transparency of the sample or operational context, transparency and appropriateness of the measurement or data-collection method, transparency of the analysis or theoretical derivation and direct relevance to remote or distributed maritime operations. Each dimension was classified as clearly reported, partially reported, not reported or not applicable. For the full evidence-characterization framework, see Appendix Table A7.

## 6 Results

Three major fields are identified in which team interaction is applied and conceptualized in the context of remote operations. This synthesis provides a robust framework for assessing collaborative “teamness” using structural and network-based measures, offering a foundation for future empirical research on hybrid human-technology teams. The major domains are shown in Table 3. Furthermore, each of these domains includes several subdomains, further specifying and illustrating the cross-disciplinary nature of the findings. These results demonstrate that although team cognition is a heavily human-focused concept, it has also been applied in more technical fields. The findings reflect a good representation of team cognition across many scientific fields. However, the thematic analysis also uncovered areas of uneven methodological implementation of the concepts.

Firstly, cognitive and psychological sciences are the most represented domains in this review. The reason for this representation is likely found in the fact that these domains include foundational research on situation awareness (Barzantny and Bruder, 2020; Demir et al., 2019; Grimm et al., 2018; Jones et al., 2021) as represented within theme one, collective cognition (Demir et al., 2019; Mathieu et al., 2022; Resick et al., 2010) represented in theme two and individual factors (Michailovs et al., 2025) as represented in theme four. Most of the studies synthesized in this literature are fundamentally psychological or cognitive investigations. Moreover, they are often validating a new measurement metric, such as check-all-that-applies [CATA] (Mathieu et al., 2022). Additionally, shared mental model networks (Resick et al., 2010) or different interventions based on behavioral science, like team training (Lacerenza et al., 2018).

Secondly, application and operational domains are represented through the focus on high-risk or complex contexts, and the analysis of the “extreme teams” within those contexts (Power, 2018) like maritime operations, naval contexts and submarines. The following is the thematic analysis of the literature.

### 6.1 Thematic analysis of the literature

Thematically, the literature ranges from situation awareness, its measurement and modelling, collective team cognition learning and training, human autonomy teaming [HAT], and remote collaboration, visualization, and interface design for shared cognition, to maritime and distributed cognition. The five themes were identified through an iterative deductive-inductive thematic analysis following Braun and Clarke’s (2006) six-phase procedure. For this review, the deductive anchor was the distributed cognition paradigm (Hutchins, 1995; Stanton, 2014), along with the interactive team cognition approach (Cooke et al., 2013). Together, they position team cognition as an emergent, system-level phenomenon distributed across human operators and technical artefacts. Within this theoretical framework, initial coding captured recurring constructs (e.g., shared mental models, situation-awareness measures, trust in automation, interface-mediated coordination, control-room configurations). Sequentially, these were grouped into candidate themes based on three criteria: (a) shared theoretical lineage, (b) similarity in unit of analysis, and (c) the operational context studied (e.g., simulated control rooms, live maritime exercises). In short, this deductive-inductive hybrid ensured that the final set of five themes is both grounded in established theory and responsive to the content of the reviewed literature.

For this review, cross-disciplinary representation of the themes was important. This stems from an effort to understand the impact of team cognition and the state of the art in applying this cognitive concept within a more technical scientific domain. Cross-disciplinary fields represented in the reviewed literature are, in order of prevalence: cognitive and psychological sciences (*n* = 40), operational domains (e.g., maritime, naval, nuclear, emergency) (*n* = 35), and engineering disciplines (e.g., network analysis and automation/AI) (*n* = 30). The prevalence does not equal the total number of articles, since most of the literature spans multiple domains. In Tables 5, a representation of fields of research and their prevalence to specific themes can be viewed.

**Table 5:** Domains and contributions.

Domain	Contribution	Articles (*n* = 51)	%. of total *n* = 51
Cognitive and psychological sciences	Individual/team cognition, measurement theory, and experimental validation.	*N* = 40	Approx. 78%
Application and operational domains	Context-specific analysis, high-stakes environments, and domain-specific challenges.	*N* = 35	Approx. 69%
Engineering disciplines	Technical systems, design methodologies, and interface evaluation.	*N* = 30	Approx. 59%

Classifications were non-exclusive. Percentages therefore sum to more than 100%. Cots indicate the number of included publications coded to each domain and do not represent mutually exclusive groups. The sum of percentages (206%) exceeds 100% due to a high degree of interdisciplinarity.

Domains were coded according to the primary disciplinary contribution and operational application of each publication. A publication could be assigned to more than one domain when it made a substantive contribution across disciplinary or methodological boundaries. For example, a publication could be coded as both an operational domain study and an engineering study when it examined a real or simulated control-room context while also evaluation a technical system or interface. Domain counts therefore represent overlapping classification rather than mutually exclusive categories. Moreover, the percentages use the total corpus of 51 included publications as the denominator and should be interpreted as descriptive indicators of disciplinary representation as opposed to estimates of the relative importance of each domain.

Moreover, there exists a significant overlap in themes and the number of articles. Following is an analysis of the five recognized themes within this literature: Situation awareness, collective team cognition, human autonomy teaming, visualization and interface design, and lastly, maritime and distributed cognition.

Shared mental models (Avnet, 2016; Floren et al., 2018), team mental models (Burtscher et al., 2011; Resick et al., 2010), and transactive memory systems (Marques-Quinteiro et al., 2013), were retained as subcodes within the broader theme of collective team cognition. This is in opposition to treating it as a separate theme. Subsequently, this decision reflects the purpose of the thematic structure, which was to organize the literature according to operational and analytical functions rather that to create a complete taxonomy of all team cognitive structures. Shared mental models primarily concern the content and similarity of team relative representations (Resick et al., 2010). Whereas, transactive memory systems concern the distribution and retrieval of specialized knowledge (Shteynberg et al., 2020; Sun et al., 2022). Both therefore contribute to the analysis of collective team cognition while remaining conceptually distinguishable within the coding framework. Their inclusion as subcodes is made explicit within the codebook in the Appendix Table A3.

Furthermore, Table 4 is an overview of thematic frequency. Frequency is only used to describe the distribution of topics within the included corpus. It does not indicate a theoretical importance, causal influence, publication quality or evidential strength. A theme represented in more publications may represent a broader searchability, disciplinary interest or conceptual overlap, rather than stronger evidence.

## 7 Theme 1: situation awareness; measurement and modeling

The first and arguably most prevalent theme is that of assessment and modeling of individual and team awareness, perception, and cognitive load. Contextualized within an operational setting, such as but not limited to remote maritime operations. Notably, the literature under review aims to operationalize how teams build and, importantly, sustain situation awareness in complex, safety-critical systems.

### 7.1 Foundational models and measurement techniques

Situation awareness has [SA] been grounded through years of research (Endsley, 1995, 1997, 2015, 2017). More precisely, through Endsley’s (1995) three-level model, focusing on the cognitive aspects of perception (level 1), comprehension (level 2), and projection (level 3). Several articles reviewed in this paper support the notion that Endsley’s three-level model is a go-to model for situation awareness (Lee et al., 2012a; Lee et al., 2012b; Lee et al., 2016; Patrick and Morgan, 2010; Saager and Harre, 2020; Zuo et al., 2020). Furthermore, the analysis reveals that maintaining SA is a cognitively taxing task. To properly examine how individuals or teams maintain SA, the concept is often exposed to hierarchical task analysis (HTA) (Patrick and Morgan, 2010). As a result, complex tasks are deconstructed into smaller, manageable actions, along with their conditions, to create a clear visual hierarchy of how SA is maintained.

Objective measurement of SA is highly individual- and task-dependent (Barzantny and Bruder, 2020; Chen et al., 2017; Michailovs et al., 2025). However, the literature points towards objective measures such as the Situation Awareness Global Assessment Scale [SAGAT] (Chen et al., 2017; Li et al., 2021). Moreover, the literature indicates a specialized objective measure that links verbal performance to specified cognitive aspects of SA called Verbal Protocol Analysis [VPA] (Lee et al., 2012b; Lee et al., 2016). Together, these methods quantify team situation awareness [TSA] either by mapping cognitive aspects to clearly specified, context-specific perceptions and planned actions (i.e., SAGAT) or to specific speech acts (VPA) (Chen et al., 2017; Lee et al., 2016; Lee et al., 2012a; Lee et al., 2012b). Furthermore, this focus highlights that awareness degradation often stems from fragmented communication and interface overload, rather than from individual cognitive deficits. The analyzed research focus on a multi-measure approach to SA (Barzantny and Bruder, 2020; Lee et al., 2016; Lee et al., 2012b; Zuo et al., 2020). Due to methodological shortcomings in the measurement of SA, it is often advised to apply a battery of measures. For example, SA measures are often coupled with performance measures, using process-tracing indicators such as eye tracking (Barzantny and Bruder, 2020). This is highly efficient in measuring cognition in control rooms or maritime bridge environments.

Furthermore, ontology-based measures are supported throughout the literature as a way of enhancing TSA in complex, high-stakes environments (Javed et al., 2011; Song et al., 2023). In Maritime Autonomous Surface Ships [MASS], the literature presents ontology-based methods to the representation of techniques that describe dependencies between human operators, displays and systems (Song et al., 2023). Here, distributed situation awareness is attempted to be achieved by unifying individual SA with authority-based SA as can be found in vessel traffic service operators [VTS]. Song et al. (2023) propose this to resolve inconsistencies based on regulatory requirements such as the Convention on the International Regulations for Preventing Collisions at Sea [COLREGs].

Taken together, recent contributions to advance SA research are allowing the field to represent team cognition as a dynamic, interdependent process rather than a uniquely individual state. This shift is somewhat paradigmatic, as previous trends have focused on the individual aspects of team-distributed SA rather than on its culmination. Moreover, the literature illustrates that the models now discussed are particularly influential in control rooms, nuclear operations, and maritime navigation because of the asynchronous data streams operators need to interpret. Finally, this points towards SA as a measurement construct and a design principle. Used properly, it can be a foundation for cognitively adaptive interfaces and workflows in remote operating environments.

The literature on SA in remote maritime and related domains converges on a central insight: Team situation awareness is not the aggregate of individual operator awareness, but a dynamic, interaction-driven phenomenon which must be measured and modelled at the system level (Endsley, 2017; Patrick and Morgan, 2010). Furthermore, the field has progressed methodologically from static freeze-probe techniques such as SAGAT (Endsley, 2017) toward network-based approaches, including situation awareness with network [SAWN] and event analysis of systemic teamwork [EAST]. These can capture the distributed flow of information across human and technical agents (Stanton, 2014; Stanton et al., 2017). Recent applications in the maritime context include ontology-based frameworks for MASS, which demonstrate the feasibility of formalizing SA reconciliation rules to manage inconsistencies between individual and authority-based SA (Song et al., 2023). However, the reliance on simulation-based freeze probe methods in a substantial pool of the reviewed literature (e.g., Barzantny and Bruder, 2020; Lee et al., 2012a; Lee et al., 2012b), remains a limitation for capturing the continuous, real-time dynamics of remote operations. Future measurements should prioritize techniques that minimize disruption to naturalistic team performance (Van Eijndhoven et al., 2023). For a presentation of team cognition measurement approaches, see Table 6. A comprehensive taxonomy of measurements is available in the Appendix Table A8.

**Table 6:** Overview of Team Cognition measurement approaches.

Measurement family and representative corpus references	Primary construct	Unit and temporal focus	Main operational value	Main limitation
Situation-awareness probes: SAGAT and related measures (Barzantny and Bruder, 2020; Lee et al., 2012a; Lee et al., 2012b)	Individual or team situation awareness.	Individual or team; event-based snapshots.	Provides structured comparison of awareness at defined decision points.	Interruptive and limited for continuous team cognition.
Network and systemic methods: SAWN and EAST (Roberts et al., 2017, 2019; Stanton, 2014; Stanton et al., 2017)	Distributed cognition, information flow, teamwork, and coordination.	Team or socio-technical system; dynamic and process-sensitive.	Captures relationships and information flow in complex operations.	Requires detailed task modelling and careful interpretation.
Protocol and text-based methods: verbal protocol analysis and CATA (Barzantny and Bruder, 2020; Lee et al., 2012a; Lee et al., 2012b; Mathieu et al., 2022)	Reasoning, sensemaking, situation awareness, and team processes.	Individual, team, or text corpus; continuous, episodic, or longitudinal.	Provides access to cognitive content and changes in team processes.	Results depend on verbalization, text quality, and coding procedures.
Shared-representation methods: shared mental models and Transactive Memory Systems (Resick et al., 2010; Shteynberg et al., 2020)	Knowledge similarity, compatibility, expertise distribution, and team learning.	Individual representations analyzed at team level; usually static or episodic.	Useful for examining cognitive structures and distributed expertise.	May obscure interaction dynamics and requires justification for aggregation.
Interaction and communication analysis (Michailovs et al., 2025; Roberts et al., 2019)	Information handoffs, coordination, adaptation, and relational structure.	Dyad, team, or network; high temporal resolution.	Well suited to cognition enacted through real-time interaction.	Observable behavior does not demonstrate cognition by itself.
Ontology, interface, and cognitive-artefact analysis (Kim et al., 2019; Song et al., 2023; Törmä et al., 2025)	Distributed situation awareness, information integration, and external cognitive support.	Socio-technical system; state-based, task-based, or comparative.	Clarifies how technologies and environments support or constrain cognition.	Interfaces and artefacts are conditions for cognition, not cognitive states themselves.

The measurement families are analytically distinct but may be combined within individual studies. This overview provides a qualitative comparison rather than a formal ranking of methodological quality. Detailed study-level applications remain in the thematic tables and Appendix Table A8.

The reviewed studies use different approaches to operationalize team cognition, situation awareness, distributed cognition and related constructs. Because these approaches address different analytical levels of temporal processes, Table 6 provides an overview of their principal uses and limitations.

The overview indicates that no single method captures the full range of team cognition. Method selection depends on whether the research question concerns cognitive structures, emergent situation awareness, interaction processes, or cognition distributed across a wider socio-technical system.

## 8 Theme 2: collective team cognition, learning and training

Collective team cognition focuses on how teams develop, share and adapt individual mental models, coordinate strategies and expertise amongst a group of people. In complex task environments, much of the empirical data lands on aligning and adapting mental models to the specific task at hand (Grimm et al., 2018; Jones et al., 2021). The current literature is moving away from static conceptualizations of shared knowledge to more dynamic, interaction-based models. As a result, the findings build on the premise that team cognition is a distributed system property that emerges from communication, shared representations, and feedback loops.

### 8.1 Models of collective cognition

Shared mental models have proven to be a prevalent concept within collective team cognition. However, recent literature has shown that measuring shared mental models must capture the underlying structures of cognition (Resick et al., 2010). For measurement, structural networks demonstrate predictive validity for team adaptation, successfully distinguishing between the similarity of a mental model and its quality (Resick et al., 2010). Previously, mental models were ranked through priority and importance, however research found this to lack the utility of similarity and quality (Resick et al., 2010).

Furthermore, collective learning theory is established as a central theory for team cognition (Shteynberg et al., 2020). Within this theory, collective attention serves as a psychological indicator for common knowledge within a given environment, which might lead to cognitive alignment between team members, further resulting in social cohesion (Mathieu et al., 2022; Shteynberg et al., 2020). Cognitive alignment will have an epistemological function, while social cohesion will be a motivational function. Together, this might facilitate superior group problem-solving functionalities and foster strategic cooperation (Mathieu et al., 2022; Shteynberg et al., 2020). However, advancing team theory proves no easy task (Kozlowski, 2015). The reviewed literature shows that this requires conceptualizing traditional static processes as dynamic and multilevel phenomena that emerge from member interactions (Kozlowski, 2015; Lacerenza et al., 2018; Mathieu et al., 2022). This necessitates other more intensive longitudinal research methods than the traditional self-reports (Kozlowski, 2015).

### 8.2 Group synergies and learning mechanisms

Previous research has shown that groups, as decision-making units, often outperform individuals. Additionally, the reviewed literature supports this by showing that groups outperform individuals in dynamic system control tasks (Schultze et al., 2021). The performance gain is often explained by the aggregation of mental models (Avnet, 2016; Avnet and Weigel, 2013; Kimmerle et al., 2011; Shteynberg et al., 2020). According to Schultze and colleagues, statistical aggregation might account for the majority of the performance gain in groups. Furthermore, true synergy effects caused by group interaction, that being group-to-individual, is a source for long-lasting acquisition of transferable knowledge regarding the systems in which groups might operate (Schultze et al., 2021).

### 8.3 Team development interventions

Alongside supporting the notion that teamwork improves decision-making, the literature also provides evidence for evidence-based interventions to improve teamwork (Lacerenza et al., 2018). Furthermore, these interventions can be categorized into training interventions and process interventions. Training interventions are straightforward in that they involve team training, leadership training, and general practice in preparation for functioning as a team (Lacerenza et al., 2018). Process interventions involve processes within a team that might increase their cooperation, such as team building and team debriefing, to further emphasize shared knowledge (Lacerenza et al., 2018). As a result, these interventions might enhance team communication, coordination, trust, and in healthcare, the reduction of fatal mistakes. Moreover, it has been shown that teams benefit from behavioral guidelines (Kimmerle et al., 2011). Such guidelines are externally provided and have been shown to be a useful instrument for overcoming individual differences in social dilemmas. Behavioral guidelines, of a high enough level, attempt to foster positive environments and clear expectations. In teams, they foster psychological safety, respect, accountability, and a sense of purpose. As a result, the team is provided with a strong “why” and core principles to guide their trajectory. Furthermore, these guidelines can be individualized to improve self-monitoring, promote goal-setting, and social support to drive change within a team.

The reviewed literature positions collective team cognition as an emergent property arising from recurrent patterns of interaction among team members, rather than as static knowledge structures residing within individuals (Cooke, 2015; Grand et al., 2016). Moreover, such an interactionist perspective is supported by empirical work demonstrating that team performance is better predicted by communication dynamics and coordination behaviors than by the sum of individual mental models (Cooke et al., 2013; Gorman, 2014). Within the maritime domain, studies of submarine control room teams have shown that reconfiguring spatial layouts and communication protocols can significantly alter information flow and shared cognition, thereby illustrating the interdependence between team processes and the physical environment (Roberts et al., 2019, 2021). Furthermore, leaning into the concept of “teamness” and the degree to which a collection of individuals functions as an integrated cognitive unit represents a promising direction in remote operation centers, where team boundaries may be fluid (Cooke et al., 2024). Additionally, transactive memory systems, wherein team members develop specialized knowledge of who knows what, further support the distributed nature of collective cognition in these settings (Shteynberg et al., 2020; Sun et al., 2022). Table 6 presents theoretical models of collective team cognition identified in the reviewed literature.

## 9 Theme 3: human autonomy teaming (HAT) and remote collaboration

Human autonomy teaming [HAT] and remote collaboration address the challenges of coordination, trust, and collaboration in systems involving both human and autonomous agents. Moreover, the field of aviation has produced extensive literature on the differences between human-only and human-autonomy teams. The literature analyzed in this review on HAT and remote collaboration points to remotely piloted aircraft systems and to fundamental differences between HATs and human-only teams.

Team coordination and team situational awareness is proven somewhat difficult in HAT’s (Demir et al., 2019). This is often attributed to coordination deficits in HATs (Demir et al., 2019). When measuring performance in coordinated awareness of situation by teams [CAST], HAT ‘score significantly lower (Demir et al., 2019). This can be attributed to the synthetic agents’ lack of anticipation and coordination, especially during unexpected events, or so-called roadblocks. Moreover, research illustrates that HAT’s also struggle with reorganizing their systems in the face of novel challenges (Grimm et al., 2018). Altogether, this can indicate a structural rigidity not present in human-only teams (Grimm et al., 2018). Despite several shortcomings, design principles for cognitive assistance systems [COGAS] in navy ships attempt to introduce flexible automation in HATs (Ozyurt et al., 2013). Consequently, this introduces flexibility by allowing the system to adapt its level of support (manual, assisted, semi-automated, or automated) based on the operator’s estimated workload and intent (Ozyurt et al., 2013). To summarize, synergizing synthetic agents and humans to achieve flexible team cognition is no easy task and requires several considerations. To further complicate HAT’s, perception and trust also play a big role in this synergy.

### 9.1 Perception and trust

The way humans perceive an autonomous agent significantly impacts team outcomes. Firstly, the cooperation is dependent on the perceived artificiality of the synthetic agent (Schelble et al., 2023). By using a “Wizard of OZ” methodology, where human operators are teamed up with what they believe to be AI, but in reality, is another human operator, Schelble et al. (2023), demonstrated that the perception of a teammate as artificial negatively impacted subjectively perceived team cognition. Moreover, the perception of negative team cognition is still prevalent even when shared mental model aggregation is not significantly affected (Schelble et al., 2023). Moreover, the literature indicates that human operators’ perception of artificiality is moderated by task difficulty (Schelble et al., 2023). This might suggest a bias or a placebo, based on prior expectations towards the capabilities of the AI (Schelble et al., 2023). Consequently, the literature points to factors other than the sharedness of one’s mental models for achieving team cognition. However, there is still the issue of trust.

Establishing trust between a synthetic agent and a human is challenging yet necessary. Furthermore, these challenges require structural and regulatory support systems (Lynch et al., 2023; Törmä et al., 2025). To achieve this, maritime autonomous surface vessels [MASS] need regulations. An example of such can be the application of Rasmussen’s risk management framework [RMF], or social network analysis [SNA] (Lynch et al., 2023). The analyzed literature illustrates that the resilience of the sociotechnical systems on MASS is positive, as it can increase the influence on regulators, as well as improve the overall resilience of the system (Lynch et al., 2023). This is important because there is sometimes a discrepancy between the pilotage and shared intent in HATs.

### 9.2 Coordination deficits and structural rigidity

The discrepancy between pilotage and intent arises from the absence of informal or in-person cues during remote operation. Systematic failures can occur due to synthetic agents’ lack of flexibility in the face of novel or ambiguous situations, as seen within distributed maritime environments (Grimm et al., 2018; Lynch et al., 2023). Such a coordination deficit is consequential in remote maritime operations, seeing how time delays and reduced communication bandwidths can amplify the gap between automated system responses and operator expectations (Demir et al., 2019). Törmä et al. (2025) attempt to bridge this gap by successfully enhancing shared situation awareness. Using a 3D route-briefing prototype, they provide a common visual reference point (Törmä et al., 2025). As a result, shared situational awareness was enhanced by clarifying navigational intent during the master-pilot exchange, in which necessary navigational information is shared (Törmä et al., 2025).

### 9.3 Cognitive assistance and adaptive automation

A natural next step is to look at cognitive assistance and adaptive automation. This sub-theme explores system architecture designed to support rather than replace human cognition. Herein, are human-in-the-loop machine learning, and adaptive task allocation (Mosqueira-Rey et al., 2023; Ozyurt et al., 2013). Interestingly, the perceived artificiality of a synthetic team member moderates these effects. As such, human performance declines when operators perceive their artificial teammates lacking in intentionality or adaptive capability (Schelble et al., 2023). See Table 7 for a presentation of core constructs within the HAT literature and each sub-theme’s key findings.

**Table 7:** Human-autonomy literature by sub-theme.

Sub-theme	Core construct	Key finding	Key reference(s)
Perception and trust	Trust calibration, automation bias	Over-reliance degrades out-of-loop performance	Cummings (2017), Strauch (2018), and Endsley (2017)
Coordination deficits	Structural rigidity, anticipation failure	Synthetic agents fail to anticipate human intent in non-routine events	Grimm et al. (2018), Demir et al. (2019), and Lynch et al. (2023)
Cognitive assistance	Adaptive automation, human-in-the-loop	Dynamic function allocation reduces workload without SA loss	Ozyurt et al. (2013), Chen et al. (2017), Mosqueira-Rey et al. (2023), and Saager and Harre (2020)

Sub-themes, and the reviewed literature’s core findings.

Syntheses of the human-autonomy literature reveals a recurring coordination deficit with the synthetic agent. As a result, synthetic agents fail to anticipate human intent during unexpected or non-routine events (Demir et al., 2019; Grimm et al., 2018). Thus, a particular deficit can be consequential in remote maritime operations, where time delays and reduced communication bandwidth amplify the gap between automated system responses and operator expectations (Lynch et al., 2023; Strauch, 2018). Moreover, empirical studies of human autonomy teams indicate that maintaining shared cognition requires active calibration of both trust and workload distribution (Chen et al., 2017; Endsley, 2017). Furthermore, the perceived artificiality of a team member moderates these effects, with studies showing that human performance declines when operators perceive their automated counterparts lacking in human tendencies such as intentionality or adaptive capability (Schelble et al., 2023). To address these challenges, the literature advocates for an adaptive automation architecture that dynamically allocates functions based on operator states and task demands (Ozyurt et al., 2013; Saager and Harre, 2020). Table 8 provides key findings from the literature reviewed.

**Table 8:** Theoretical models of collective team cognition.

Model	Emergence mechanism	Temporal dynamics	Operationalization	Key reference(s)
Interactive team cognition	Interaction patterns	Real-time, dynamic	Communication network analysis	Cooke (2015) and Cooke et al. (2013)
Transactive memory systems	Knowledge specialization	Stable over time	Survey, recall accuracy	Shteynberg et al. (2020) and Sun et al. (2022)
Teamness	Degree of integration	Variable, context-dependent	Coordination behavior coding	Cooke et al. (2024)

Collective team cognition, their theoretical models and how the reviewed literature measured each concept.

## 10 Theme 4: visualization and interface design for shared cognition

Visualization and interface design for shared cognition examines how interface designs, visual encoding, data fusion and cognitive ergonomics influence awareness and coordination. To start, the analyzed literature indicates that physical and digital configurations are major determinants of communication efficiency.

### 10.1 Designing for team communication

Control room layouts are optimal when they move away from engineering constraints and move toward human-centered layouts. For example, Stanton and colleagues (2020) introduced a novel inward-facing, circular configuration for submarine control rooms. This indicated that the volume of verbal communication decreased significantly. However, compared with more traditional layouts, network cohesion and task completion were significantly increased (Stanton et al., 2022). Furthermore, developing digital fire control centers on ships using human-centered design [HCD] enhances shared situation awareness (Petermann et al., 2024). This results from integrating fire plans, sensor status, and stowage data into a single interface (Petermann et al., 2024). As a result, communication gaps are mitigated, while response times are reduced (Petermann et al., 2024). Additionally, active group-view displays are preferred over passive group-view displays (Kim et al., 2019). This was investigated in nuclear power plants, where active displays featuring real-time pointing and marking functions were shown to significantly improve shared situation awareness between operator pairs (Kim et al., 2019). Consequently, this suggests that interface designs directly mitigate hazards towards team cognition and reduce error hazards (Kim et al., 2019). One approach is to reduce clutter in overview displays (Mikkelsen et al., 2012). Guidelines, as mentioned by Mikkelsen and colleagues, suggest that displays should favor discrete color coding for critical limits and use non-color variables for nominal states. Furthermore, high-salience colors should be reserved for alarms (Mikkelsen et al., 2012). As such, operators can easily, and without too much cognitive effort, distinguish between alarms, critical limits and normal states.

### 10.2 Information integration

For team performance, managing information flow is critical. This is because the flow of information across a visual interface can increase shared cognition. By achieving high information integration, meaning the extent to which one can make critical data widely available via consoles, there is also a chance that one can improve team situation awareness (Michailovs et al., 2025). The research suggests that, with information readily available, teams can focus more on refining solutions rather than categorizing the information presented (Michailovs et al., 2025). However, when considering the human aspects in this larger sociotechnical system, the analyzed literature shows that military voice protocols can restrict the emergence of structural social information networks (Michailovs et al., 2025). As a consequence, this finding might show that protocol overrides purely technological affordances in relation to information integration (Michailovs et al., 2025).

To further information integration, urban telepresence can be applied. These systems aim to provide superhuman abilities in regard to situation awareness, overview and comprehension (Balfour and Donnelly, 2013). This is achieved by providing live, past and predictive data into a 4D augmented reality browser (Balfour and Donnelly, 2013). As a result, remote operators are able to maintain a comprehensive spatial–temporal understanding of distant environments (Balfour and Donnelly, 2013). Consequently, providing enough information to enable a clear overview of past, current and future situations.

The reviewed literature consistently confirms that interface design for remote maritime operations must shift from supporting individual data accessibility to enabling distributed sensemaking across a team (Cort and Lindblom, 2025; Törmä et al., 2025). Consequently, single-operator displays are insufficient for team-based supervisory control, where multiple operators must integrate heterogeneous data streams to construct a shared operational picture. As such, tools that externalize navigational intent, such as 3D route-briefing prototypes for remote pilotage, are essential for bridging the gap created by the absence of co-located communication cues from informants (Törmä et al., 2025). Studies of passive group-view displays and shared situation awareness suggest that common operational pictures can improve team situation awareness; however, only when they are designed to support both divergent and convergent interpretations (Kim et al., 2019; Mikkelsen et al., 2012). Moreover, the increasing role of augmented reality and smart glasses in control room environments further opens the potential to overlay cognitive artefacts directly onto the operator’s field of view. Thereby, reducing the need for effortful information retrieval (Lim et al., 2018; Petermann et al., 2024).

## 11 Theme 5: maritime and distributed cognition

The theme of maritime and distributed cognition is primarily context- and domain-specific. As such, it addresses the application of distributed cognition theories and human-system interaction specifically within naval and maritime operational settings (Table 9).

**Table 9:** Interface design function and supporting evidence.

Design function	Key finding	Reference(s)
Shared sensemaking displays	Interface must support distributed sensemaking across the team, not individual data access	Cort and Lindblom (2025) and Törmä et al. (2025)
3D route briefing prototypes	Externalizing navigational intent bridges the gap from missing co-located cues	Törmä et al. (2025)
Passive group-view displays	Common operational pictures improve team SA when supporting divergent and convergent interpretations	Kim et al. (2019); Mikkelsen et al. (2012)
Augmented reality / smart glasses	Overlaying cognitive artefacts onto operator’s field of view reduces effortful information retrieval	Lim et al. (2018); Petermann et al. (2024)

Supporting evidence for different design functions.

### 11.1 Distributed cognition as an analytical framework

For analyzing work in complex systems, distributed cognition is proven to be the primary theoretical lens (Lynch et al., 2023; Nilsson et al., 2012; Stanton, 2014). Moreover, for analyzing information fusion, that being the integration of information arriving from different sources, distributed cognition is necessary as it views human operators as active. Nilsson et al. (2012), argue that human operators are active participants in the information fusion process (Nilsson et al., 2012). Expressed simply, the human operator cooperates with the technology to achieve level two within the Joint Directors of Laboratory [JDL] models for data fusion. In level two, operators achieve situation assessment, where they interpret relationships between objects and events in any given context (Nilsson et al., 2012). Distributed cognition models cognition as distributed across people, technological installations, and the general environment. By tracing how information is propagated and transformed through people, technological artefacts and the wider system, researchers can obtain observable indicators of cognitive processes (Nilsson et al., 2012). These indicators do not constitute cognition by themselves. Rather their interpretation requires evidence concerning task relevance, information transformation and consequences for collective understanding or action.

### 11.2 Submarine command and control

The Event Analysis of Systemic Teamwork [EAST], a network analysis framework, is applied in multiple of the analyzed studies (Roberts et al., 2017, 2019; Stanton et al., 2017, 2021). This is also primarily applied in the simulated Royal Navy submarine control room.

In high-demand operations, bottlenecks need to be identified. Several of the analyzed articles center on specific bottlenecks around specific systemic parts. For instance, returning to periscope depth, or inshore operations, creates communication and workload bottlenecks, specifically surrounding operation officers and sonar controllers (Roberts et al., 2017, 2019; Stanton et al., 2017). With these bottlenecks identified, design implementations can be adapted around these. Moreover, design interventions, such as co-location and circular configurations, have been shown to significantly improve team performance (Roberts et al., 2021; Stanton et al., 2021). By reducing communication volume and transferring responsibility for communication, communicative cohesion increased, further improving team performance and efficiency.

Visualizing is important in both shallow and deep waters. During certain submarine operations, periscope data takes a more central role than passive sonar data. Conversely, this centrality can make shallow-water navigation difficult, as shallow waters require greater passive sonar engagement (Stanton, 2014). This may reflect a trend toward sensors dominating information channels (Michailovs et al., 2025). Moving further up towards the surface, bearing, the horizontal angle between the direction of a vessel and a reference point, remains the most critical cue for predicting vessel movement.

### 11.3 Remote situatedness and socio-technical resilience

When working remotely, accessing and engaging with the physical world can be challenging. Especially considering that many of the physical cues to which one is accustomed either do not exist or are simulated by artificial haptic feedback. As such, the literature indicates that, to achieve remote situatedness, railway traffic operators manage it by way of integrating tacit situated knowledge with mediated views from analogue artefacts. As such, a meaningful engagement with a distant physical location is achieved (Cort and Lindblom, 2025). The importance of this is also evident in the information that is lost when physical proximity is not feasible. For example, technology’s inability to convey the physical constraints in the world (train stopping on an incline) can lead to operator and system failures (Cort and Lindblom, 2025). Such physical attributes are also relevant regarding “ship sense,” which can be lost when working remotely.

Even though MASS is a sociotechnical system in growth, it can be argued that it is growing more quickly than is advisable. As such, MASS requires validated system-based risk management and social network analysis. This is apparent, seeing how MASS is accelerating at an increasing rate, but the gap between the regulation of Remote-Control Centres (RCC) and the necessary knowledge, understanding, and proficiency [KUP] for remote personnel is still large (Lynch et al., 2023). In short, the technical environment is advancing faster than operators’ knowledge and understanding. Considering this, Lynch et al. (2023) identified, through a social network analysis, that industry and regulators are the most central and influential actors in this socio-technical environment. Through cooperation, these can narrow the gap, furthering remoteness by advancing technology, while keeping personnel along.

For this theme, the reviewed literature operationalizes the distributed cognition perspective directly in maritime operational contexts. In submarine command and control, studies demonstrate that alterations to control room configuration produce measurable effects on communication efficiency, information flow and team coordination (Roberts et al., 2017, 2021; Stanton et al., 2017). The literature suggests simply changing from traditional layouts to circular arrangements (Roberts et al., 2017, 2021; Stanton et al., 2017). See Table 10 for a presentation of distributed cognition studies in the maritime context. Furthermore, studies of reduced crew sizes and remote operation centers suggest that distributed cognition frameworks are particularly valuable for predicting how the removal of co-located interaction channels affects team performance (Roberts et al., 2019; Veitch and Alsos, 2024). In the emergency management domain, ontology-based inference systems have been developed to enhance team SA by formalizing the knowledge structures that underpin distributed decision making (Javed et al., 2011). The challenge of maintaining distributed SA in mixed waterborne transport, where manned vessels, autonomous ships, and shore-based operators must coordinate, highlights the need for frameworks that explicitly model SA as a reconciliatory process across multiple actors (Song et al., 2023).

**Table 10:** Distributed cognition studies in the maritime context.

Study focus	Operational context	Key finding	Reference(s)
Submarine control room configuration	Submarine C2	Circular layout alters communication efficiency and information flow	Roberts et al. (2017, 2021) and Stanton et al. (2017)
Reduced crewing and remote operations	Submarine / remote ops centers	Removal of co-located interaction channels degrades team performance	Roberts et al. (2019) and Veitch and Andreas Alsos (2024)
Ontology-based SA for emergency management	Emergency management	Formalized inference systems enhance team distributed SA	Javed et al. (2011)
Distributed SA in mixed waterborne transport	MASS / manned vessels	SA must be modelled as a reconciliatory process across multiple actors	Song et al. (2023); Stanton et al. (2006)

The maritime context of distributed cognition studies for this review.

## 12 Discussion on thematic support

To conclude this thematic analysis, the findings are now applied the maritime incidents introduced earlier in the review. The analyzed results can be used to understand real-world incidents, such as the USS Fitzgerald and Helge Ingstad incidents. Both accidents should be understood as failures of an entire distributed cognitive system, rather than as individual human errors (Theme 2) (Avnet, 2016; Avnet and Weigel, 2013). The crew had sufficient cognitive artefacts (radar, AIS, displays) that contained sufficient data, but the teams never transformed this data into shared, actionable mental models (Theme 4) (Resick et al., 2010; Zhang et al., 2022). Alongside this, the operators of this incident might have gotten decision inertia, and fallen prey to the confirmation bias, seeing how the pressure of imminent collision might have forced their thinking into trusting initial assessment and ignoring disconfirming evidence (Theme 3) (Kim et al., 2011) Applying EAST and identifying bottlenecks, as in previous studies by Roberts et al. (2017, 2019) and Stanton et al. (2017), could be extremely helpful. For instance, identifying bottlenecks that might have slowed communication and led to the incidents could help prevent them. This is likely due to a mismatch between expected communication links and the sparse network that existed at the time of the collision (Fincannon et al., 2011). Further, this might have led to a decline in shared situation awareness (Theme 1). Lastly, both vessels use a traditional control room layout in which operators are physically separated. This is a high-risk factor, as it can create barriers to productive communication and can lead to information overload (Kim et al., 2019). Table 10 provides a side-by-side comparison of how these themes apply to the incidents.

Furthermore, team performance should not be treated as a direct or interchangeable measure of team cognition. Performance is an outcome, as such it may be influenced by several factors. For example, team cognition might absolutely play a role in performance, however, so can expertise, redundancy, task difficulty, automation support, equipment reliability available resources, staffing and environmental conditions. Conversely, a team may display effective information sharing, adaptive cooperation and compatible situation awareness, while performing poorly due to equipment failures, external disturbances, insufficient time or constraints outside the teams control. The present review therefore distinguishes between cognitive structures such as shared mental models, cognitive processes, such as interactive information transformation, behavioral processes such as communication and coordination, and contextual affordances such as interface design and performance outcomes. Claims about team cognition should not be inferred from performance alone (Table 11).

**Table 11:** Mapping of maritime incidents to reviewed themes.

Theme	USS Fitzgerald (2017)	Helge Ingstad (2018)
Situation awareness	Inconsistent SA between the watch team and OOD regarding collision risk	Conflicting SA between the bridge team and VTS regarding traffic separation
Collective team cognition	Absence of shared mental model; watch team did not challenge OOD decisions	Lack of shared understanding of navigation plan; deviation from standard protocols
Human-autonomy teaming	Over-reliance on AEGIS system; alarm fatigue	Insufficient automation feedback on navigation mode
Visualization and interface design	Cluttered tactical displays; critical information not salient	Poor integration of AIS data with bridge team’s visual scan
Maritime and distributed cognition	Information not propagated across the watch team hierarchy	Communication breakdown between VTS and vessel; distributed information not reconciled

Application of themes to maritime incidents.

The body of literature analyzed in this review (*n* = 51) provides an extensive overview of situation awareness and its modelling (Theme 1). Furthermore, shared mental models are analyzed to understand the team’s collective cognition, learning, and training (Theme 2). Moreover, the literature proposes approaches to mitigate automation costs through teaming and remote collaboration (Theme 3) and to validate novel interfaces that aid both individual and team cognition (Theme 4). Lastly, the literature examines distributed cognition in remote work (Theme 5). For a condensed overview of themes, the number of articles corresponding with each theme, and sub-topics, see Table 12. For a full list of articles, their corresponding themes and justification for inclusion, see the Appendix.

**Table 12:** Quantified theme composition.

Theme	Count	Key sub-topics
SA/M	32	Measurement tools (SAGAT, SPAM, CAST), cognitive load assessment, team SA modelling, Endsley’s three-level model, verbal protocol analysis
CTC/L/T	31	Shared mental models, team learning cycles, coordination mechanisms, training interventions, transactive memory systems, and team debriefing
HAT/RC	12	Human-AI teaming, synthetic agents, UAV/UGV control, remote collaboration, supervisory control, automation bias, trust calibration
V/ID	11	3D visualization, power system displays, interface design principles, information integration, Ecological Interface Design, display clutter
M/DC	19	Submarine operations, maritime autonomy, distributed cognition, control room configurations, sensor fusion, nautical safety, VTS operations

Articles cross-listed, which influence the total count. SA/M = Situation awareness, measurement and modelling. CTC/L/T = Collective Team Cognition, Learning and Training. HAT/RC=Human-autonomy teams and remote cognition. V/ID=Visualization and interface design. M/DC = Maritime and distributed cognition.

Additionally, the literature addresses these themes with multi-methodological support, while the maritime operational domain remains a rich contextual backdrop. To summarize the analyzed literature and its corresponding themes: system-level, dynamic analysis is necessary. This is to fully understand how team cognition emerges from the complex interactions among people, technology, and procedures. To further emphasize this point, Table 13 can be viewed to get a sense of the co-occurrence of themes.

**Table 13:** Heatmap of co-occurring articles within themes.

Themes	M/DC	HAT/RC	V/ID	SA/M	CTC/L/T
M/DC	19	3	3	8	8
HAT/RC	3	12	2	7	3
V/ID	3	2	11	5	4
SA/M	8	7	5	32	17
CTC/L/T	8	3	4	17	31

Heatmap of articles belonging to several themes. CTC/L/T = Collective Team Cognition, Learning and Training. HAT/RC=Human-autonomy teams and remote cognition. V/ID=Visualization and interface design. M/DC = Maritime and distributed cognition. Green- High co-occurence, Red - low co-occurence.

Noticeably, the distribution of cognition amongst human agents and technological implementations is important. The literature indicates that team cognition in multi-operator tasks is not achievable without necessary interventions, both among human operators and between human operators and the technology they interact with. Moreover, the primary cognitive product of this interaction, which also influences operating behavior, is situation awareness. Situation awareness is discussed several times in this review. Therefore, it is necessary to understand its individual, distributed and operationalized properties. Clearly, situation awareness is widely regarded as a cornerstone of safe operations across several domains. This holds true for individuals and teams as well. Results derived from this analysis can have far-reaching implications. Most crucially, this research has implications for designers, trainers and regulators.

## 13 Implications

Firstly, designers should adopt a human-centered approach, focusing on information-centric interface layouts. For example, designs should shift away from engineering-driven console placements toward configurations that promote direct information flows among interdependent operators (Roberts et al., 2021). Furthermore, shared views and interactive displays, such as active group displays that allow real-time pointing, marking, and annotation, can dramatically improve shared situation awareness and cut response times compared to passive displays (Mikkelsen et al., 2012). Interfaces should also serve as external memory and reasoning aids, using attentive visual cues and highlighting the relevance of information to the current task (Mathieu et al., 2022). Alongside this, cognitive artefacts (radar, AIS, sonar) should support distributed cognition. Moreover, automation should be designed to be transparent and adjustable based on the operator’s workload (Chen et al., 2017). With automations come challenges in mitigating teammate artificiality in order to maintain good team cognition (Schelble et al., 2023). Lastly, designers must take into account remote situatedness and support this by embedding cues that preserve a sense of place (Cort and Lindblom, 2025). Examples include 3D visualization and augmented reality overlays.

Secondly, trainers must shift their focus from individual factors to team-process training (Kim et al., 2019; Lee et al., 2016; Lee et al., 2012b). These programs should develop collective mental models, shared situation awareness, and coordination capabilities, rather than only individual agent task proficiency. Furthermore, in order to maintain high-quality communication and trust, debriefings organized around performance and teamwork-related categories, in combination with psychological safety practices, should be applied (Lacerenza et al., 2018). Moreover, training must include explicit decision points for when to engage or disengage with automation and, more importantly, how to recover situational awareness after prolonged use of automated support (Cort and Lindblom, 2025). Conclusively, training should also focus on how operators can develop metacognitive skills (Michailovs et al., 2025). As such, operators must learn to recognize when they have become a communication bottleneck, and when to request workload redistribution before performance degrades (Michailovs et al., 2025).

Third and last, regulators should mandate system-based risk management and socio-technical oversight. Regulations should require social network analysis, ontology-based modelling, or EAST-type network analysis to identify critical decision-makers, communication bottlenecks, and other issues before systems are deployed (Nazir et al., 2012; Nazir et al., 2014). Staffing should also be linked to human information processing (Wickens, 2002). Within this framework, perception and cognition function as interconnected processes that enable humans to interpret sensory input and make decisions. Match this with a workload analysis (NASA-TLX), and regulators can increase staffing levels in response to increased cognitive load (Lin et al., 2013). Standards can also be set for performance-based interfaces. Certification can be used to verify that interfaces support distributed cognition (e.g., active group-view displays) (Kim et al., 2019). In HAT’s regulators can ensure transparent and adaptive automation. They can ensure agents proactively provide critical information rather than waiting for operator requests (Schelble et al., 2023). Furthermore, establishing clear terminology for human teammates, AI teammates, and automation levels could reduce ambiguity in liability. This can, in turn, ensure that perceived artificiality does not undermine general safety (Schelble et al., 2023). Lastly, continuous monitoring of situation awareness could be enforced. Periodically, team situation awareness (SAGAT, CAST, and verbal-protocol analysis) could become a natural part of the audit cycle to uphold the three levels of the situation awareness hierarchy under real-world conditions (Lee et al., 2012a; Lee et al., 2012b).

The findings in this review connect to a substantial body of maritime human factors research examining team coordination in real-world operational contexts. For example, studies of joint activity in the maritime traffic systems have shown that ship masters, maritime pilots, tug masters, and VTS operators develop distinct situational models that must be actively reconciled for safe navigation (Mansson et al., 2017). Such examples align directly with the distributed cognition and situation awareness themes identified in this review. Research by Porath et al. (2013) on maritime communication, as well as by Grech and Lemon (2008) on situation awareness in shipping, further underscores the importance of shared mental models in bridge teams, supporting the collective team cognition theme. Similarly, work by Praetorius and Lützhöft (2012), on VTS operator cognition and by Øvergård et al. (2015), on navigational decision-making provides empirical grounding for the human autonomy teaming and interface design themes discussed in this review.

## 14 Limitations

Like most literature reviews, this is also a snapshot in time. As such, it is recognized that relevant literature outside the domains and time period covered in this review might provide further insight into the challenges of establishing team cognition in complex environments. Moreover, this review aimed to investigate team cognition in remote maritime operations; however, research in that area is somewhat limited. As such, literature from neighboring domains, such as submarine operations, aviation, and emergency responders, is included. The reasoning for this is that they also include sentiments from team cognition, and by proxy, humans in complex, uncertain and critical occupations. By reviewing literature deemed to be relevant to the problem at hand, this has shed light on where the research within remote maritime operations need to focus going forward. Integrating this literature more closely with the cognitive engineering frameworks analyzed here represents a productive direction for future work.

A limitation concerns the reproducibility of the EBSCOhost search. The original search was conducted on December 16th, 2025 using the EBSCOhost collections and field structure available at the time. The platform and database configuration have since changed, and the current interface does not reproduce the collection, indexing or field environment exactly. Although the archived search string and 62-recorded EBSCOhost export were retained, the current platform reconstruction produced a different set of records. Consequently, the current sensitivity comparison can describe the effect of the additional conceptual block under the present platform configuration. However, it cannot establish counterfactual effect on the original 62-record search. This limitation is reported transparently, and the archived search export remains the authoritative source for the reviews original study-selection process.

The evidence-characterization framework was not intended to constitute a conventional risk-of-bias assessment or a validated methodological quality scale. It characterizes reporting transparency, methodological information, construct measurement, analytical derivation and transferability across a heterogeneous evidence base. It therefore informed interpretation and qualification of the synthesis but did not estimate conventional risk of bias, internal validity or overall study quality. The framework should not be interpreted as equivalent to a formal risk of bias instrument.

## 15 Future directions

A systematic extraction of future research recommendations across the reviewed studies reveals three overarching priorities.

First, longitudinal validation of network-based measurement techniques such as EAST, SAWN, and computer-aided text analysis is needed within real remote operations centers in order to establish their sensitivity to changes in team cognition over time (Cooke et al., 2013; Gorman et al., 2020; Mathieu et al., 2022).

Second, intervention protocols such as team debriefings alongside cross-training and adaptive automation strategies should be empirically tested in HAT’s specific to maritime contexts, drawing on evidence-based frameworks from team development research (Lacerenza et al., 2018; Nazir et al., 2014).

Third, comparative studies quantifying the cognitive and communicative differences between traditional bridge crews and remote operators are necessary. These can inform domain-specific regulatory frameworks and future training standards (Kari and Steinert, 2021).

Future research should focus on measuring individual-level cognitive variables and upscale them to the team level. Importantly, research must also distinguish between constructs that can legitimately be modelled as shared team properties and processes that are enacted through interaction. Aggregation from individual responses to the team level is appropriate only when the theoretical model treats the construct as a shared emergent state and when empirical evidence supports aggregation. Future studies should therefore report within-team agreement, and between-team variability, alongside a clear theoretical justification for the decision to aggregate. Aggregation is more suitable for constructs such as shared mental models or shared situation awareness when team members demonstrate sufficient compatibility in their representations. It is less suitable for dynamic interaction processes, such as information handoffs, communication sequences, coordination adaptations or distributed cognitive work. For these processes, relational, sequential or network-based approaches such as EAST, Sawhn, interaction coding and temporal network analysis, are more appropriate due to their preservation of the timing and structure of interactions.

Moreover, maritime operations are a constantly evolving environment, and although much research has been conducted across a variety of maritime settings, more research is needed in specific settings, such as remote maritime operations. This should be done to get a clearer picture of how team cognition might affect remote operations differently from traditional operations. Additionally, there is research applicable to different domains. At this point, there should be some intervention designs to sustain team cognition in remote maritime operations. Especially to mitigate declines in team cognition when operators fall out of the loop. These need to be tested and retested longitudinally; however, they offer a tangible opportunity to increase operators’ effectiveness in maritime settings. First and foremost, however, there must be research comparing the differences in operators’ cognition, both individually and in teams, between remote operators and traditional operators.

## 16 Conclusion

In conclusion, this review underscores that team cognition is not merely a supplementary factor but a foundational requirement for safe remote maritime operations. The literature reveals that in high-stakes, sociotechnical systems, cognition is an emergent property. It is distributed across human operators, technical artefacts/implementations and environmental constraints. However, applying these distributed processes in practice remains the field’s primary challenge. To advance from theoretical modelling to large-scale implementation, the field must embrace systematic, network-based measurement techniques such as EAST and CATA. As a result, one might capture the dynamic ebb and flow of team processes in real time. Future maritime safety will depend on the field’s collective ability to design, test and validate these sociotechnical interactions. As a result, one might ensure that the systems in place support the human operator’s active, integrative role, rather than isolating them through fragmented workflows. By redesigning workspaces and interfaces to support distributed cognition, training teams to operate as a cohesive cognitive unit, and regulating at the level of sociotechnical systems rather than their isolated components, the maritime domain can close the gaps that contribute to incidents such as those involving the USS Fitzgerald and the Helge Ingstad. Further moving toward a safer, more resilient form of maritime operations.

## Data Availability

The original contributions presented in the study are included in the article/Supplementary material, further inquiries can be directed to the corresponding author/s.
