# Supplementary material for: Team cognition in remote maritime operations: a scoping review and cross-domain theoretical synthesis
Source: Front Psychol. 2026 Oct 2;17:1843838. doi: 10.3389/fpsyg.2026.1843838 (PMC13672215; doi:10.3389/fpsyg.2026.1843838)
Supplement: Supplementary file 1 [file Supplementary_file_1.docx]

**Appendix**

**Table A1.**

Title and Author Year Mapping Connected to Primary Themes

**Theme mapping (n=51)**

| **#** | **Title** | **Primary Theme(s)** | **Thematic Justification** |
| --- | --- | --- | --- |
| **1** | (Lin et al., 2013)*. Analyzing the staffing and workload in the main control room of the advanced nuclear power plant from the human information processing perspective* | **SA/M, CTC/L/T** | Directly measures workload, staffing, and human information processing stages (perception, cognition, response) correlated with operator roles |
| **2** | (Chen et al., 2017)*. Optimizing the balance between task automation and human manual control in simulated submarine track management* | **SA/M, HAT/RC** | Investigates automation levels, situation awareness (SAGAT), and manual control regain in human-machine systems |
| **3** | (Resick et al., 2010)*. Mental model metrics and team adaptability: A multi-facet multi-method examination* | **CTC/L/T, SA/M** | Examines shared mental models, team adaptation, and performance using network-based structural approaches |
| **4** | (Kimmerle et al., 2011)*. Value of information, behavioral guidelines, and social value orientation in an information-exchange dilemma* | **CTC/L/T** | Studies team information sharing, coordination, and how behavioral guidelines override individual differences in collaborative contexts |
| **5** | (Shteynberg et al., 2020)*. Shared worlds and shared minds: A theory of collective learning and a psychology of common knowledge* | **CTC/L/T, SA/M** | Proposes theory of collective learning and common knowledge formation, directly relevant to team cognition |
| **6** | (Schelble et al., 2023)*Investigating the Effects of Perceived Teammate Artificiality on Human Performance and Cognition* | **HAT/RC, CTC/L/T** | Directly examines human-AI teaming, shared mental models, and perceived team cognition differences |
| **7** | (Mathieu et al., 2022)*. Indexing Dynamic Collective Constructs Using Computer-Aided Text Analysis: Construct Validity Evidence and Illustrations Featuring Team Processes* | **CTC/L/T, SA/M** | Focuses on team processes, dynamic constructs, and text analysis for team cognition |
| **8** | (Avnet, 2016)*. A Network‐Based Analysis of Team Coordination and Shared Cognition in Systems Engineering* | **CTC/L/T, SA/M** | Uses network analysis of coordination, shared mental models, and team learning in engineering teams |
| **9** | (Lacerenza et al., 2018)*. Team development interventions: Evidence-based approaches for improving teamwork* | **CTC/L/T** | Comprehensive review of team training, debriefing, and team building interventions |
| **10** | (Kozlowski & Chao, 2018)*. Unpacking team process dynamics and emergent phenomena: Challenges, conceptual advances, and innovative methods* | **CTC/L/T, SA/M** | Discusses team process dynamics, emergent states, and innovative measurement methods |
| **11** | (Power, 2018)*. Extreme teams: Toward a greater understanding of multiagency teamwork during major emergencies and disasters* | **CTC/L/T, M/DC** | Focuses on emergency team coordination, communication, cooperation in multi-agency contexts |
| **12** | (Avnet & Weigel, 2013)*. The Structural Approach to Shared Knowledge* | **CTC/L/T** | Proposes methodology for analyzing shared knowledge networks in teams |
| **13** | (Lynch et al., 2023)*. The application of a system‐based risk management framework and social network analysis to the Maritime Autonomous Surface Ship system: Who are the decision‐makers in the wider system?* | **M/DC, HAT/RC** | Analyzes maritime autonomy, distributed decision-making, and socio-technical systems |
| **14** | (Stanton et al., 2017)*. Up periscope: understanding submarine command and control teamwork during a simulated return to periscope depth* | **M/DC, SA/M** | Submarine control room study using EAST method for team SA and communication |
| **15** | (Roberts et al., 2017)*. Land Ahoy! Understanding Submarine Command and Control During the Completion of Inshore Operations* | **M/DC, SA/M** | Submarine team performance, social/information/task networks, distributed SA under varying demands |
| **16** | (Li et al., 2021)*. An experimental study on the effects of task complexity and knowledge and experience level on SA, TSA and workload* | **SA/M, CTC/L/T** | Measures individual/team SA, workload, and effects of task complexity and operator experience |
| **17** | (Patrick & Morgan, 2010)*. Approaches to understanding, analysing and developing situation awareness* | **SA/M, CTC/L/T** | Proposes task-oriented systems perspective and process tracing methodology for SA analysis |
| **18** | (Roberts et al., 2019)*. The effects of team co-location and reduced crewing on team communication characteristics* | **CTC/L/T, M/DC** | Submarine team study on co-location effects, communication networks, and workload |
| **19** | (McNeese & Cooke, 2016)*. Team Cognition as a Mechanism for Developing Collaborative and Proactive Decision Support in Remotely Piloted Aircraft Systems* | **HAT/RC, SA/M** | Investigates team cognition, human-autonomy teaming, and decision support in RPAS |
| **20** | (Petermann et al., 2024) *Collaborative Hazard Control: Designing a Digital Fire Control Center for Enhanced Safety on Ships* | **V/ID, M/DC** | Human-centered design of digital fire control interface for maritime safety |
| **21** | (Zuo et al., 2020)*. Experimental Research on Measurement of Team Situation Awareness in Nuclear Power Plants* | **SA/M, CTC/L/T** | Develops team SA measurement scale and methods for nuclear plant operations |
| **22** | (Roberts et al., 2021)*It's a circular argument: Examining how a novel configuration impacts information flow in submarine control rooms* | **M/DC, CTC/L/T** | Novel circular submarine control room configuration, information flow, team process |
| **23** | (Schultze et al., 2021)*. A test of synergy in dynamic system control tasks* | **SA/M, CTC/L/T** | Experimental test of group vs. individual performance in dynamic system control |
| **24** | (Stanton, 2014)*Representing distributed cognition in complex systems: how a submarine returns to periscope depth* | **M/DC, SA/M** | EAST method applied to submarine operations, task/social/information networks |
| **25** | (Song et al., 2023)*Distributed Situational Awareness for Maritime Autonomous Surface Ships in Mixed Waterborne Transport: An Ontology-based Framework* | **M/DC, HAT/RC, SA/M** | Maritime autonomy, distributed SA framework, ontology-based modeling for MASS |
| **26** | (Stanton et al., 2022)*. The quest for the ring: a case study of a new submarine control room configuration* | **M/DC, CTC/L/T** | Inward-facing ring configuration for submarine control rooms, team process changes |
| **27** | (Barzantny & Bruder, 2020)*. Measuring situation awareness of operation teams in NPPs using a verbal protocol analysis* | **SA/M, CTC/L/T** | Verbal protocol analysis method for measuring team SA in nuclear power plants |
| **28** | (Jones et al., 2021)*. Construction of Shared Situational Awareness in Traffic Management* | **SA/M, CTC/L/T, M/DC** | Ethnographic study of traffic management center operators building shared SA |
| **29** | (McKendrick et al., 2011)*. Team Performance and Communication within Networked Supervisory Control Human-Machine Systems* | **HAT/RC, SA/M** | Human supervisory control over networked unmanned systems, teamwork |
| **30** | (Cort & Lindblom, 2025)*. Remote situatedness beyond the control room: enhanced seeing and sense of place in railway operations* | **M/DC, SA/M** | Railway operations, remote situational awareness, distributed cognition |
| **31** | (Fincannon et al., 2011)*. Team Size, Team Role, Communication Modality, and Team Coordination in the Distributed Operation of Multiple Heterogeneous Unmanned Vehicles* | **HAT/RC, CTC/L/T** | Team size, roles, communication modalities in UV operations |
| **32** | (Stanton & Roberts, 2020)*. Block off: an examination of new control room configurations and reduced crew sizes examining engineered production blocking* | **M/DC, CTC/L/T** | Submarine control room configuration changes, crew size reduction impacts |
| **33** | (Javed et al., 2011)*. Ontology-Based Inference to Enhance Team Situation Awareness in Emergency Management* | **SA/M, CTC/L/T** | Ontology-based approach to enhancing team SA in emergency contexts |
| **34** | (Stanton et al., 2021)*Returning to periscope depth in a circular control room configuration* | **M/DC, CTC/L/T** | Circular submarine control room design, communication efficiency, task completion |
| **35** | (Salman Nazir et al., 2014)*. How distributed situation awareness inlfuences process safety* | **M/DC, SA/M** | Distributed situation awareness in process industry safety |
| **36** | (Ozyurt et al., 2013)*. Simulation-Based Development of a Cognitive Assistance System for Navy Ships* | **V/ID, HAT/RC** | Cognitive assistance system design for naval combat information centers |
| **37** | (McLane et al., 2010)*. Concept Analysis of Cognitive Artifacts* | **CTC/L/T, V/ID** | Analysis of cognitive artifacts in socio-technical systems |
| **38** | (Kim et al., 2011)*. Development and validation of a heuristic model for evaluation of the team performance of operators in nuclear power plants* | **CTC/L/T, SA/M** | Heuristic model for team performance evaluation in nuclear plants |
| **39** | (Grimm et al., 2018)*Team Situation Awareness in Human-Autonomy Teaming: A Systems Level Approach* | **HAT/RC, SA/M** | Systems-level analysis of team SA in human-autonomy teams |
| **40** | (Kim et al., 2019)*. Empirical Study of Shared Situation Awareness between Active and Passive Group-view Displays* | **SA/M, V/ID** | Comparison of active vs. passive group displays for shared SA |
| **41** | (Balfour & Donnelly, 2013)*. The What, Why and How of Achieving Urban Telepresence* | **HAT/RC, SA/M, V/ID** | Urban telepresence system for enhanced situational awareness and overwatch missions |
| **42** | (Lee et al., 2016)*. Measuring Situation Awareness of Operating Team in Different Main Control Room Environments of Nuclear Power Plants* | **SA/M, V/ID** | Measures team SA in digital vs. conventional nuclear plant control rooms |
| **43** | (Mikkelsen et al., 2012)*. Visualization of Power System Data on Situation Overview Displays* | **V/ID, SA/M** | Information visualization for shared situation awareness in power grid control |
| **44** | (Lee et al., 2012)*. Measuring situation awareness of operation teams in NPPs using a verbal protocol analysis* | **SA/M, CTC/L/T** | Verbal protocol analysis method for measuring team SA in nuclear plants. |
| **45** | (Promann et al., 2016)*. The role of aesthetics and perception in raising situation awareness* | **SA/M, CTC/L/T, V/ID** | User-centered information visualization design concept for large screens in network operations centers. Aims to raise situation awareness by visualizing large scale network data. |
| **46** | (Demir et al., 2019). *Team coordination of team situation awareness in human-autonomy teaming* | **SA/M, CTC/L/T, HAT/RC** | Explicit TSA focus in HAT´s and all-human teams in a simulated Remote Piloted Aircraft System task environment. |
| **47** | (Michailovs et al., 2025)*. The effect of information integration on team communication in a simulated submarine control room task* | **M/DC, V/ID, CTC/L/T** | Information integration impact on team communication and distributed SA in submarine simulation. |
| **48** | (Törmä et al., 2025)*. Enhancing Shared Situational Awareness in Remote Pilotage: A Prototype for 3D Route Briefing* | **V/ID, M/DC** | 3D visualization prototype for remote pilotage and shared SA enhancement. |
| **49** | (Nilsson et al., 2012)*. Information fusion in practice: A distributed cognition perspective on the active role of users* | **M/DC, HAT/RC** | Maritime surveillance case study using distributed cognition framework for information fusion. |
| **50** | (Saager & Harre, 2020)*. Modeling distributed situational awarareness to improve handling emergency calls in operation centers* | **SA/M, M/DC** | Presents a model called DSA-HEC (Distributed Situational Awareness for Handling Emergency Calls), in order to understand DSA in operation centers. |
| **51** | (Power & Alison, 2017)*. Redundant deliberation about negative consequences: Decision inertia in emergency responders* | **CTC/L/T, V/ID** | Applied critical decision methods to major emergencies, and their responders to reduce decision inertia. |

*Note.* Reviewed articles and their correspondence with the themes analysed. Following with a thematic justification.

**Theme Composition Summary**

| **Theme** | **Count** | **Key Sub-Topics** |
| --- | --- | --- |
| **SA/M** | 32 | Measurement tools (SAGAT, SPAM, CAST), cognitive load assessment, team SA modeling, Endsley's three-level model, verbal protocol analysis |
| **CTC/L/T** | 31 | Shared mental models, team learning cycles, coordination mechanisms, training interventions, transactive memory systems, team debriefing |
| **HAT/RC** | 12 | Human-AI teaming, synthetic agents, UAV/UGV control, remote collaboration, supervisory control, automation bias, trust calibration |
| **V/ID** | 11 | 3D visualization, power system displays, interface design principles, information integration, Ecological Interface Design, display clutter |
| **M/DC** | 19 | Submarine operations, maritime autonomy, distributed cognition, control room configurations, sensor fusion, nautical safety, VTS operations |

*Note.* Some articles are cross-listed under multiple themes where their content significantly addresses more than one conceptual area. The total counts reflect these overlaps.

**Codebook**

**Table A2.**

Theme 1 – Situation Awareness; Measurement and Modelling

| Dimension | Description |
| --- | --- |
| Definition | Research on the assessment, modeling, and validation of individual and team situation awareness (SA/TSA) in operational environments. |
| Analytical focus | Measurement tools (e.g., SAGAT, SPAM), ontological modeling, cognitive load, and shared awareness formation. |
| Sub-codes | SA Measurement; TSA Assessment; Cognitive Load; Control Room Awareness; Ontological Modeling. |
| Inclusion criteria | Papers developing or applying frameworks to quantify awareness, perception, or projection in team or control settings. |
| Exclusion criteria | Studies addressing awareness as a secondary outcome of training or interface design (coded under Themes 2 or 4). |
| Illustrative papers | Barzantny & Bruder (2020); Lee et al. (2016); Li et al. (2021). |
| Key concepts | Endsley’s Model of SA; team SA; cognitive alignment; dynamic systems modeling. |

**Table A3.**

Theme 2 – Collective Team Cognition, Learning, and Training

| Dimension | Description |
| --- | --- |
| Definition | Studies exploring how teams develop, share, and adapt mental models and coordination strategies. |
| Analytical focus | Team learning, collective cognition, adaptive training design, and psychological safety. |
| Sub-codes | Shared Mental Models; Team Training; Adaptive Expertise; Learning Feedback; Cognitive Coordination, Shared Mental Models, Transactive Memory Systems, Team Mental Models |
| Inclusion criteria | Research explicitly addressing team-level cognition, coordination, or learning processes. |
| Exclusion criteria | Papers focusing primarily on individual awareness metrics or interface design. |
| Illustrative papers | Grimm et al. (2018); Jones et al. (2021); Lacerenza et al. (2018). |
| Key concepts | Collective cognition; team learning cycles; coordination theory; cross-monitoring. |

**Table A4.** Theme 3 – Human Autonomy Teaming (HAT) and Remote Collaboration

| Dimension | Description |
| --- | --- |
| Definition | Research addressing coordination, communication, and trust between humans and autonomous systems in shared tasks. |
| Analytical focus | Automation bias, transparency, adaptive autonomy, and shared intent in human–machine systems. |
| Sub-codes | Trust Calibration; Automation Bias; Communication Grounding; Adaptive Autonomy; Human–AI Collaboration. |
| Inclusion criteria | Studies involving joint human–machine performance, remote control of autonomous systems, or supervisory decision-making. |
| Exclusion criteria | Research on human-only teamwork or interface visualization without autonomy components. |
| Illustrative papers | Demir et al. (2019); McNeese & Cooke (2016); Cooke et al. (2020). |
| Key concepts | Human–autonomy trust; transparency; mutual predictability; dynamic authority allocation. |

**Table A5.**

Theme 4 – Visualization and Interface Design for Shared Cognition

| Dimension | Description |
| --- | --- |
| Definition | Studies on how interface design, visualization, and information integration influence cognition and coordination. |
| Analytical focus | Visual analytics, data fusion, user-centered display design, and perceptual ergonomics. |
| Sub-codes | Interface Visualization; Data Fusion; Cognitive Ergonomics; Shared Displays; Information Ecology. |
| Inclusion criteria | Papers focusing on visual, auditory, or multimodal interface design for improving awareness or coordination. |
| Exclusion criteria | Conceptual papers without empirical or design-oriented focus. |
| Illustrative papers | Kim et al. (2019); Mikkelsen, Johansson, & Cooper (2016); Jones et al. (2021). |
| Key concepts | Cognitive artifacts; visualization transparency; perceptual salience; design for shared SA. |

**Table A6.**

Theme 5 – Maritime and Distributed Cognition

| Dimension | Description | |
| --- | --- | --- |
| Definition | Domain-specific research examining cognition distributed across humans, tools, and systems in maritime contexts. | |
| Analytical focus | Distributed cognition, remote bridge operations, MASS supervision, and socio-technical system resilience. | |
| Sub-codes | | Distributed Cognition; Remote Bridge Control; Maritime Autonomy; Socio-Technical Systems; Cognitive Resilience. |
| Inclusion criteria | Papers focused on maritime or oceanic contexts, addressing human–system interaction or distributed control. | |
| Exclusion criteria | Non-maritime studies or those without distributed cognition relevance. | |
| Illustrative papers | Stanton et al. (2020); Li et al. (2021); Johnson et al. (2022). | |
| Key concepts | Distributed cognition; remote operations; MASS; cognitive coupling; socio-technical integration. | |

**Table A7.**

Operational theme-to-criterion

**Appraisal criteria.** C1 = purpose and design transparency; C2 = context and evidence-source transparency; C3 = construct and measurement transparency; C4 = analysis or theoretical-derivation transparency; C5 = directness and transferability to remote maritime operations.

| **Theme code** | **Primary criteria** | **Reason for emphasis** |
| --- | --- | --- |
| SA/M | C2, C3, C4, C5 | Context, measurement, analytical modelling, and transferability are central to situation-awareness studies. |
| CTC/L/T | C1, C3, C4, C5 | These studies require attention to purpose, construct operationalisation, theoretical or analytical derivation, and operational transferability. |
| HAT/RC | C1, C2, C3, C5 | Human-autonomy and remote-collaboration studies require scrutiny of design, operational context, measures, and transferability. |
| V/ID | C2, C3, C5 | Interface studies require scrutiny of context, visualisation or measurement procedures, and relevance to remote operations. |
| M/DC | C2, C4, C5 | Distributed and maritime cognition studies require scrutiny of system context, analytical derivation, and maritime transferability. |

This matrix reports the evidence-characterisation ratings and justification for all 51 included publications. C1 through C5 represent reporting transparency, methodological information, construct and measurement transparency, methodological, analytical or theoretical derivation and directness and transferability. These ratings are not traditional risk-of-bias scores.

**Corpus matrix**

| **No.** | **Publication** | **Theme code(s)** | **Thematic justification** | **Primary appraisal criteria** | **C1/C2/C3/C4/C5** | **Evidence-characterization note** |
| --- | --- | --- | --- | --- | --- | --- |
| 1 | 'Lin et al. (2013 | SA/M; CTC/L/T | Directly measures workload, staffing, and human information processing stages (perception, cognition, response) correlated with operator roles | C1-C5 | C/C/C/C/P | Empirical ANPP control-room study; explicit questionnaire, CFA, reliability and statistical analyses; non-maritime transferability. |
| 2 | 'Chen et al. (2017 | SA/M; HAT/RC | Investigates automation levels, situation awareness (SAGAT), and manual control regain in human-machine systems | C1-C5 | C/C/C/C/P | Three simulated submarine experiments with named SA, workload and automation measures; transfer to remote maritime operations remains indirect. |
| 3 | 'Resick et al. (2010 | CTC/L/T; SA/M | Examines shared mental models, team adaptation, and performance using network-based structural approaches | C1-C5 | C/C/C/C/P | Multi-method team experiment with explicit participants, tasks, mental-model metrics and adaptation analysis; non-maritime transferability. |
| 4 | 'Kimmerle et al. (2011 | CTC/L/T | Studies team information sharing, coordination, and how behavioral guidelines override individual differences in collaborative contexts | C1, C3, C4, C5 | C/C/C/C/P | Two information-exchange experiments with participants, conditions, dependent contribution measure and ANOVA; indirect operational transfer. |
| 5 | 'Shteynberg et al. (2020 | CTC/L/T; SA/M | Proposes theory of collective learning and common knowledge formation, directly relevant to team cognition | C1-C5 | C/P/NA/C/P | Theory paper with a clear collective-learning argument; no empirical operational sample or measurement; maritime transfer is conceptual. |
| 6 | 'Schelble et al. (2023 | HAT/RC; CTC/L/T | Directly examines human-AI teaming, shared mental models, and perceived team cognition differences | C1-C5 | C/C/C/C/P | Controlled HAT experiment with participants, simulated task, mental-model measures and statistical tests; maritime transfer is indirect. |
| 7 | 'Mathieu et al. (2022 | CTC/L/T; SA/M | Focuses on team processes, dynamic constructs, and text analysis for team cognition | C1-C5 | C/C/C/C/P | Methodological CATA study with multiple samples, coding protocol and validity analyses; transfer to maritime operations is indirect. |
| 8 | 'Avnet (2016 | CTC/L/T; SA/M | Uses network analysis of coordination, shared mental models, and team learning in engineering teams | C1-C5 | C/C/C/C/P | Engineering design sessions, surveys, network measures and correlation analysis are described; the setting limits generalisability. |
| 9 | 'Lacerenza et al. (2018 | CTC/L/T | Comprehensive review of team training, debriefing, and team building interventions | C1, C3, C4, C5 | C/P/NA/C/P | Evidence-based review with an explicit intervention focus; no original sample or empirical measures; maritime transfer is conceptual. |
| 10 | 'Kozlowski and Chao (2018 | CTC/L/T; SA/M | Discusses team process dynamics, emergent states, and innovative measurement methods | C1-C5 | C/P/NA/C/P | Conceptual and methodological article that clearly discusses team-process dynamics and methods; no primary sample or measurement. |
| 11 | 'Power (2018 | CTC/L/T; M/DC | Focuses on emergency team coordination, communication, cooperation in multi-agency contexts | C1-C5 | C/P/NA/C/P | Conceptual review of extreme teams and multi-agency teamwork; empirical context varies and maritime transfer is indirect. |
| 12 | 'Avnet and Weigel (2013 | CTC/L/T | Proposes methodology for analyzing shared knowledge networks in teams | C1, C3, C4, C5 | C/C/C/C/P | Engineering design methodology with design sessions, survey-based shared-knowledge measures, DSM and correlation analysis; not maritime. |
| 13 | 'Lynch et al. (2023 | M/DC; HAT/RC | Analyzes maritime autonomy, distributed decision-making, and socio-technical systems | C1-C5 | C/C/C/C/C | Maritime autonomous surface-ship system study with explicit socio-technical, risk and network analysis; directly relevant. |
| 14 | 'Stanton et al. (2017 | M/DC; SA/M | Submarine control room study using EAST method for team SA and communication | C2, C3, C4, C5 | C/C/C/C/C | Submarine simulation with team sample, scenarios, EAST networks and performance and communication measures; direct maritime relevance. |
| 15 | 'Roberts et al. (2017 | M/DC; SA/M | Submarine team performance, social/information/task networks, distributed SA under varying demands | C2, C3, C4, C5 | C/C/C/C/C | Submarine inshore-operations study with explicit operational context, teams, network measures and analysis; direct maritime relevance. |
| 16 | 'Li et al. (2021 | SA/M; CTC/L/T | Measures individual/team SA, workload, and effects of task complexity and operator experience | C1-C5 | C/C/C/C/P | NPP experiment explicitly measures SA, TSA and workload under task complexity and experience; transfer is indirect. |
| 17 | 'Patrick and Morgan (2010 | SA/M; CTC/L/T | Proposes task-oriented systems perspective and process tracing methodology for SA analysis | C1-C5 | C/P/NA/C/P | Methodological and theoretical SA article with a task-oriented perspective and process tracing; no primary empirical maritime sample. |
| 18 | 'Roberts et al. (2019 | CTC/L/T; M/DC | Submarine team study on co-location effects, communication networks, and workload | C1-C5 | C/C/C/C/C | Submarine study with co-location, communication networks and workload, with clear design and analysis; direct maritime relevance. |
| 19 | 'McNeese and Cooke (2016 | HAT/RC; SA/M | Investigates team cognition, human-autonomy teaming, and decision support in RPAS | C1-C5 | C/C/C/C/P | RPAS team-cognition and decision-support study with an explicit application; transfer to maritime operations is indirect. |
| 20 | 'Petermann et al. (2024 | V/ID; M/DC | Human-centered design of digital fire control interface for maritime safety | C2, C3, C4, C5 | C/C/P/P/C | Maritime fire-control design and user study; purpose and context are clear, but the small sample and early-stage evaluation limit measurement and analytical completeness. |
| 21 | 'Zuo et al. (2020 | SA/M; CTC/L/T | Develops team SA measurement scale and methods for nuclear plant operations | C1-C5 | C/C/C/C/P | NPP team-SA measurement study with defined measures and validation against performance; transfer is indirect. |
| 22 | 'Roberts et al. (2021 | M/DC; CTC/L/T | Novel circular submarine control room configuration, information flow, team process | C1-C5 | C/C/C/C/C | Submarine control-room configuration study with explicit scenarios, network measures and statistical comparison; direct maritime relevance. |
| 23 | 'Schultze et al. (2021 | SA/M; CTC/L/T | Experimental test of group vs. individual performance in dynamic system control | C1-C5 | C/C/C/C/P | Three-experiment dynamic-control study with explicit sample, design and analysis; non-maritime transferability. |
| 24 | 'Stanton (2014 | M/DC; SA/M | EAST method applied to submarine operations, task/social/information networks | C2, C3, C4, C5 | C/C/C/C/C | Submarine distributed-cognition and EAST study with explicit operational system and network modelling; direct maritime relevance. |
| 25 | 'Song et al. (2023 | M/DC; HAT/RC; SA/M | Maritime autonomy, distributed SA framework, ontology-based modeling for MASS | C1-C5 | C/C/P/C/C | MASS ontology framework with explicit maritime scope and model derivation; measurement validation is limited. |
| 26 | 'Stanton et al. (2022 | M/DC; CTC/L/T | Inward-facing ring configuration for submarine control rooms, team process changes | C1-C5 | C/C/C/C/C | Submarine ring-configuration case study with serving submariners, scenarios, communication and task measures, and EAST analysis; direct maritime relevance. |
| 27 | 'Barzantny and Bruder (2020 | SA/M; CTC/L/T | Verbal protocol analysis method for measuring team SA in nuclear power plants | C1-C5 | C/C/C/C/P | NPP verbal-protocol measurement study with a defined method and performance comparison; transfer is indirect. |
| 28 | 'Jones et al. (2021 | SA/M; CTC/L/T; M/DC | Ethnographic study of traffic management center operators building shared SA | C1-C5 | C/C/C/C/P | Traffic-management ethnography with observations and interviews, defined analysis and situated context; maritime transfer is indirect. |
| 29 | 'McKendrick et al. (2011 | HAT/RC; SA/M | Human supervisory control over networked unmanned systems, teamwork | C1-C5 | C/C/C/C/P | Networked supervisory-control experiment with explicit participants, task, factors and performance measures; transfer is indirect. |
| 30 | 'Cort and Lindblom (2025 | M/DC; SA/M | Railway operations, remote situational awareness, distributed cognition | C2, C3, C4, C5 | C/C/P/C/P | Railway workplace and ethnographic study with clear context and qualitative method; construct measurement and maritime transfer are limited. |
| 31 | 'Fincannon et al. (2011 | HAT/RC; CTC/L/T | Team size, roles, communication modalities in UV operations | C1-C5 | C/C/C/C/P | Distributed unmanned-vehicle experiment with sample, team-size and modality manipulations, process measures and ANOVAs; transfer is indirect. |
| 32 | 'Stanton and Roberts (2020 | M/DC; CTC/L/T | Submarine control room configuration changes, crew size reduction impacts | C1-C5 | C/C/C/C/C | Submarine configuration and reduced-crew study with explicit sample, scenarios, network data and statistical analysis; direct maritime relevance. |
| 33 | 'Javed et al. (2011 | SA/M; CTC/L/T | Ontology-based approach to enhancing team SA in emergency contexts | C1-C5 | C/C/P/C/P | Ontology-based emergency-management framework; purpose, context and derivation are clear, but empirical measurement validation is limited. |
| 34 | 'Stanton et al. (2021 | M/DC; CTC/L/T | Circular submarine control room design, communication efficiency, task completion | C1-C5 | C/C/C/C/C | Submarine circular-configuration study with scenarios, communication and task outcomes, and statistical analysis; direct maritime relevance. |
| 35 | 'Salman Nazir et al. (2014 | M/DC; SA/M | Distributed situation awareness in process industry safety | C2, C3, C4, C5 | C/C/P/C/P | Process-industry distributed-SA study with explicit safety context and conceptual and measurement linkage; maritime transfer is limited. |
| 36 | 'Ozyurt et al. (2013 | V/ID; HAT/RC | Cognitive assistance system design for naval combat information centers | C1, C2, C3, C5 | C/C/P/P/C | Naval cognitive-assistance simulation and design study; maritime context is clear, but initial design and evaluation evidence is limited. |
| 37 | 'McLane et al. (2010 | CTC/L/T; V/ID | Analysis of cognitive artifacts in socio-technical systems | C1-C5 | C/P/NA/C/P | Concept analysis of cognitive artefacts with clear conceptual derivation; no primary operational sample or empirical measurement. |
| 38 | 'Kim et al. (2011 | CTC/L/T; SA/M | Heuristic model for team performance evaluation in nuclear plants | C1-C5 | C/C/P/C/P | Nuclear team-performance heuristic model with an explicit development pathway; measurement and generalisation limitations remain. |
| 39 | 'Grimm et al. (2018 | HAT/RC; SA/M | Systems-level analysis of team SA in human-autonomy teams | C1-C5 | C/C/P/C/P | HAT systems-level paper with explicit conceptual framing and application; construct measurement and maritime transfer are indirect. |
| 40 | 'Kim et al. (2019 | SA/M; V/ID | Comparison of active vs. passive group displays for shared SA | C2, C3, C4, C5 | C/C/C/C/P | Nuclear control-room display experiment with active and passive conditions, SACRI and operational experts; transfer is indirect. |
| 41 | 'Balfour and Donnelly (2013 | HAT/RC; SA/M; V/ID | Urban telepresence system for enhanced situational awareness and overwatch missions | C1-C5 | C/P/P/P/P | Urban telepresence paper with a clear system aim but limited direct evidence and a non-maritime setting; construct and analysis detail are limited. |
| 42 | 'Lee et al. (2016 | SA/M; V/ID | Measures team SA in digital vs. conventional nuclear plant control rooms | C2, C3, C4, C5 | C/C/C/C/P | Nuclear control-room comparison with simulator, verbal-protocol TSA measures and performance linkage; transfer is indirect. |
| 43 | 'Mikkelsen et al. (2012 | V/ID; SA/M | Information visualization for shared situation awareness in power grid control | C2, C3, C4, C5 | C/C/P/P/P | Power-grid field studies across multiple control rooms using cognitive task analysis; visualisation design and analytic detail are partial, and the setting is non-maritime. |
| 44 | 'Lee et al. (2012 | SA/M; CTC/L/T | Verbal protocol analysis method for measuring team SA in nuclear plants | C1-C5 | C/C/C/C/P | NPP verbal-protocol study with an explicit method for team SA and analysis; transfer is indirect. |
| 45 | 'Promann et al. (2016 | SA/M; CTC/L/T; V/ID | User-centered information visualization design concept for large screens in network operations centers. Aims to raise situation awareness by visualizing large scale network data | C1-C5 | C/C/C/C/P | SpringRain visualisation study with explicit design rationale and perceptual and SA focus; maritime transfer is indirect. |
| 46 | 'Demir et al. (2019 | SA/M; CTC/L/T; HAT/RC | Explicit TSA focus in HAT´s and all-human teams in a simulated Remote Piloted Aircraft System task environment | C1-C5 | C/C/C/C/P | HAT and RPAS study with explicit teams, task environment, communication and SA measures, and analysis; transfer is indirect. |
| 47 | 'Michailovs et al. (2025 | M/DC; V/ID; CTC/L/T | Information integration impact on team communication and distributed SA in submarine simulation | C1-C5 | C/C/C/C/C | Simulated submarine control-room study with teams, manipulated information integration, communication, SA and workload measures, and statistical analysis; direct maritime relevance. |
| 48 | 'Törmä et al. (2025 | V/ID; M/DC | 3D visualization prototype for remote pilotage and shared SA enhancement | C2, C3, C4, C5 | C/C/P/P/C | Remote-pilotage prototype with maritime professionals and semi-structured interviews; early-stage, small-sample evaluation without task-performance metrics. |
| 49 | 'Nilsson et al. (2012 | M/DC; HAT/RC | Maritime surveillance case study using distributed cognition framework for information fusion | C1-C5 | C/C/P/C/C | Maritime-surveillance case study with explicit distributed-cognition framework, observation, context and analysis; measurement is qualitative and limited. |
| 50 | 'Saager and Harre (2020 | SA/M; M/DC | Presents a model called DSA-HEC (Distributed Situational Awareness for Handling Emergency Calls), in order to understand DSA in operation centers | C2, C3, C4, C5 | C/C/P/C/P | Emergency-call DSA-HEC model with explicit scenario, observation and model-building method; empirical validation and maritime transfer are limited. |
| 51 | 'Power and Alison (2017 | CTC/L/T; V/ID | Applied critical decision methods to major emergencies, and their responders to reduce decision inertia | C1-C5 | C/C/C/C/P | Emergency-responder decision-inertia study with explicit critical-decision methods and empirical context; maritime transfer is indirect. |

**Coding rule for the completed evidence-characterization appraisal**

**C:** Clearly reported.

**P:** Partially reported.

**N:** Not reported.

**NA:** Not applicable to the publication type or study purpose.

Interpretive note. This completed matrix applies C/P/NA ratings to the 51 corpus publications. The ratings characterize reporting transparency and directness for this scoping review; they are not conventional risk-of-bias scores. C3 is coded NA for conceptual publications where empirical measurement is outside the publication’s purpose.

Table A8.

Conceptual taxonomy of central team cognitive constructs.

| **Construct** | **Definition and role in the framework** | **Primary ontological status** | **Unit or level of analysis** | **Mechanism or analytical focus** | **Typical operationalisation in the review** | **Conceptual boundary** |
| --- | --- | --- | --- | --- | --- | --- |
| Team cognition | Umbrella construct for how task-relevant knowledge is organised, exchanged, transformed, and used by a team or socio-technical system. It includes related structures, processes, and emergent states, but is not reducible to any one of them (Cooke et al., 2013; Cooke, 2015; DeChurch & Mesmer-Magnus, 2010). | Umbrella construct spanning cognitive structures, cognitive processes, and emergent team-level properties. | Team, multiteam system, or socio-technical system. | Collective use and coordination of task, role, goal, and environmental knowledge over time. | Interaction measures, communication-content and handoff measures, shared-mental-model measures, team situation-awareness measures, and network approaches (Cooke & Gorman, 2009; Gorman et al., 2020). | Not synonymous with shared mental models, team situation awareness, communication, or distributed cognition. Those are components, manifestations, indicators, or broader perspectives. |
| Shared or team mental models | Organised representations of task requirements, roles, goals, procedures, and the operational environment that are similar, compatible, or structurally related across team members (Mathieu et al., 2000; Mohammed et al., 2010). | Cognitive structure, with team-level emergence when member representations become sufficiently compatible or complementary. | Individual representations assessed in relation to the team or dyad. | Similarity, compatibility, accuracy, quality, overlap, and complementarity of task and team knowledge. | Concept mapping, paired-comparison or structured elicitation, similarity and accuracy metrics, and structural network analysis (Resick et al., 2010). | Describes the organisation or content of represented knowledge. It does not by itself demonstrate the interaction through which knowledge is developed or enacted. |
| Transactive memory system | A distributed knowledge system in which members develop specialised expertise and knowledge of who knows what, together with processes for locating and retrieving that expertise (Shteynberg et al., 2020; Marks et al., 2001). | Distributed cognitive structure plus a retrieval and coordination process. | Team or network of members, with expertise distributed across individuals. | Specialisation, directory knowledge, access, retrieval, and coordination of distributed expertise. | Knowledge-location and expertise measures, recall or recognition tasks, team-member ratings, and network representations. | Differs from a shared mental model because members need not possess the same knowledge. The shared element concerns knowledge of expertise distribution and access. |
| Team situation awareness | Team-level awareness of relevant elements of the operational situation, their meaning, and their likely development, produced through the coordination of information across actors (Endsley, 1995; Gorman et al., 2006). | Emergent team state that is maintained through an ongoing cognitive process. | Team, operational unit, or distributed system. | Perception, comprehension, and projection of task-relevant events, conditions, and future states. | SAGAT or TSAGAT-type probes, verbal-protocol analysis, CAST, SAWN, and related network or ontology-based approaches (Gorman et al., 2006; Javed et al., 2011; Song et al., 2023). | A situation-specific manifestation of team cognition. It is not equivalent to all team cognition and should not be inferred from communication frequency alone. |
| Interactive team cognition | The view that team cognition is enacted in the real-time interaction among team members, especially when interaction carries, transforms, negotiates, or coordinates task-relevant information (Cooke et al., 2013; Cooke, 2015). | Cognitive process enacted through team interaction. | Dyad, team, or interaction network. | Information exchange, interpretation, transformation, anticipation, and coordination during task performance. | Interaction coding, information-handoff measures, communication-content analysis, sequence analysis, and temporal network analysis (Cooke & Gorman, 2009; Gorman et al., 2020). | Observable interaction is not automatically cognition. Communication and coordination are treated as evidence of cognitive interaction only when task-relevant content and cognitive uptake or transformation are identifiable. |
| Distributed cognition | A broader theoretical perspective in which cognitive work is distributed across people, social interaction, artefacts, technologies, representations, and the environment (Hutchins, 1995; Stanton, 2014). | Socio-technical theoretical perspective and system-level explanatory framework. | Distributed cognitive system, including human and non-human elements. | How representations, tools, spatial arrangements, procedures, and interactions jointly accomplish cognitive work. | EAST, cognitive-work and systems analysis, network analysis, distributed situation-awareness modelling, and ontology-based modelling (Stanton, 2014; Song et al., 2023). | Broader than team cognition. It is not restricted to human team members and does not require a bounded team or shared mental representation. |
| Communication and coordination | Observable behavioural and relational processes through which team members exchange information, align activity, manage dependencies, and perform handoffs. | Behavioural or relational process, and possible mechanism or indicator of cognition. | Dyad, team, network, or system. | Timing, content, direction, sequence, centrality, handoff success, and adaptation of coordinated activity. | Communication frequency and content, information-handoff rates, network centrality, sequence measures, and coordination coding (Cooke & Gorman, 2009; Roberts et al., 2019). | These behaviours are not cognitive states in themselves. Their relevance to team cognition depends on task content, interpretation, uptake, and consequences for the team's evolving representation or action. |
| Interface, cognitive artefacts, and operational environment | External resources and environmental arrangements that make information visible, available, interpretable, or shareable, or that constrain how cognitive work is distributed (Hutchins, 1995; Song et al., 2023). | Technological or environmental affordance and contextual condition. | Human-system interface, control room, vessel, network, or wider socio-technical system. | Externalisation, integration, visibility, accessibility, spatial organisation, and distribution of information and work. | Interface evaluation, display and artefact analysis, control-room configuration comparisons, ontology-based models, and system-level case analysis (Javed et al., 2011; Roberts et al., 2019; Song et al., 2023). | These conditions support or constrain cognition but are not themselves team cognition, a mental model, or team situation awareness. |

**References**

Avnet, M. S. (2016). A Network‐Based Analysis of Team Coordination and Shared Cognition in Systems Engineering. *Systems Engineering*, *19*(5), 395–408. https://doi.org/10.1002/sys.21341

Avnet, M. S., & Weigel, A. L. (2013). The Structural Approach to Shared Knowledge: An Application to Engineering Design Teams. *Human Factors: The Journal of the Human Factors and Ergonomics Society*, *55*(3), 581–594. https://doi.org/10.1177/0018720812462388

Balfour, R. E., & Donnelly, B. P. (2013). The what, why and how of achieving urban telepresence. *2013 IEEE Long Island Systems, Applications and Technology Conference (LISAT)*, 1–6. https://doi.org/10.1109/LISAT.2013.6578234

Barzantny, C., & Bruder, C. (2020). Measuring Situation Awareness in Control Room Teams. In D. Harris & W.-C. Li (Eds), *Engineering Psychology and Cognitive Ergonomics. Cognition and Design* (Vol. 12187, pp. 3–17). Springer International Publishing. https://doi.org/10.1007/978-3-030-49183-3_1

Chen, S. I., Visser, T. A. W., Huf, S., & Loft, S. (2017). Optimizing the balance between task automation and human manual control in simulated submarine track management. *Journal of Experimental Psychology: Applied*, *23*(3), 240–262. https://doi.org/10.1037/xap0000126

Cort, R., & Lindblom, J. (2025). Remote situatedness beyond the control room: Enhanced seeing and sense of place in railway operations. *Cognition, Technology & Work*, *27*(1–2), 375–400. https://doi.org/10.1007/s10111-025-00802-z

Demir, M., McNeese, N. J., She, M., & Cooke, N. J. (2019). Team Coordination of Team Situation Awareness in Human-Autonomy Teaming. *Proceedings of the Human Factors and Ergonomics Society Annual Meeting*, *63*(1), 146–147. https://doi.org/10.1177/1071181319631259

Fincannon, T., Keebler, J. R., Jentsch, F., Phillips, E., & Evans, A. W. (2011). Team Size, Team Role, Communication Modality, and Team Coordination in the Distributed Operation of Multiple Heterogeneous Unmanned Vehicles. *Journal of Cognitive Engineering and Decision Making*, *5*(1), 106–131. https://doi.org/10.1177/1555343411399073

Grimm, D. A., Demir, M., Gorman, J. C., & Cooke, N. J. (2018). Team Situation Awareness in Human-Autonomy Teaming: A Systems Level Approach. *Proceedings of the Human Factors and Ergonomics Society Annual Meeting*, *62*(1), 149–149. https://doi.org/10.1177/1541931218621034

Javed, Y., Norris, T., & Johnston, D. (2011). *Ontology-based inference to enhance team situation awareness	in	emergency	management*.	Scopus. https://www.scopus.com/inward/record.uri?eid=2-s2.0-84905665924&partnerID=40&md5=f57961fc8defdc4a3fe1030244932116

Jones, R., Beach, M. W., McClure Haughey, M., Sutherland, W., & Lee, C. P. (2021). Construction of Shared Situational Awareness in Traffic Management. *Proceedings of the ACM on Human-Computer Interaction*, *5*(CSCW1), 1–27. https://doi.org/10.1145/3449128

Kim, S. K., Byun, S. N., & Lee, D. H. (2011). Development and validation of a heuristic model for evaluation of the team performance of operators in nuclear power plants. *Annals of Nuclear Energy*, *38*(9), 2005–2016. Scopus. https://doi.org/10.1016/j.anucene.2011.04.016

Kim, S. K., Sim, J. H., Jang, T. I., & Lee, H. C. (2019). Empirical Study of Shared Situation Awareness between Active and Passive Group-view Displays. *Proceedings of the Human Factors and Ergonomics Society Annual Meeting*, *63*(1), 2195–2200. https://doi.org/10.1177/1071181319631072

Kimmerle, J., Wodzicki, K., Jarodzka, H., & Cress, U. (2011). Value of information, behavioral guidelines, and social value orientation in an information-exchange dilemma. *Group Dynamics: Theory, Research, and Practice*, *15*(2), 173–186. APA PsycArticles (2011-07800-001). https://doi.org/10.1037/a0021467

Kozlowski, S. W. J., & Chao, G. T. (2018). Unpacking team process dynamics and emergent phenomena: Challenges, conceptual advances, and innovative methods. *American Psychologist*,	*73*(4),	576–592.	APA	PsycArticles	(2018-23205-021). https://doi.org/10.1037/amp0000245

Lacerenza, C. N., Marlow, S. L., Tannenbaum, S. I., & Salas, E. (2018). Team development interventions: Evidence-based approaches for improving teamwork. *American Psychologist*, *73*(4), 517–531. APA PsycArticles (2018-23205-017). https://doi.org/10.1037/amp0000295

Lee, S. W., Kim, A. R., Park, J., Kang, H. G., & Seong, P. H. (2016). Measuring Situation Awareness of Operating Team in Different Main Control Room Environments of Nuclear Power Plants. *Nuclear Engineering and Technology*, *48*(1), 153–163. Scopus. https://doi.org/10.1016/j.net.2015.09.008

Lee, S. W., Park, J., Kim, A. R., & Seong, P. H. (2012). Measuring situation awareness of operation teams in NPPs using a verbal protocol analysis. *Annals of Nuclear Energy*, *43*, 167–175. https://doi.org/10.1016/j.anucene.2011.12.005

Li, P., Wang, Y., Chen, J., Luo, Z., & Dai, L. (2021). An experimental study on the effects of task complexity and knowledge and experience level on SA, TSA and workload. *Nuclear Engineering and Design*, *376*, 111112. https://doi.org/10.1016/j.nucengdes.2021.111112

Lin, C. J., Yenn, T.-C., Jou, Y.-T., Hsieh, T.-L., & Yang, C.-W. (2013). Analyzing the staffing and workload in the main control room of the advanced nuclear power plant from the human information processing perspective. *Safety Science*, *57*, 161–168. Scopus. https://doi.org/10.1016/j.ssci.2013.02.004

Lynch, K. M., Banks, V. A., Roberts, A. P. J., Downes, J., Radcliffe, S., & Plant, K. L. (2023). The application of a system‐based risk management framework and social network analysis to the Maritime Autonomous Surface Ship system: Who are the decision‐makers in the wider system? *Human Factors and Ergonomics in Manufacturing & Service Industries*, *33*(5), 395–429. https://doi.org/10.1002/hfm.21000

Mathieu, J. E., Wolfson, M. A., Park, S., Luciano, M. M., Bedwell-Torres, W. L., Ramsay, P. S., Klock, E. A., & Tannenbaum, S. I. (2022). Indexing dynamic collective constructs using computer-aided text analysis: Construct validity evidence and illustrations featuring team processes. *Journal of Applied Psychology*, *107*(4), 533–559. APA PsycArticles (2021-82593-001). https://doi.org/10.1037/apl0000856

McKendrick, R., Shaw, T., Saqer, H., De Visser, E., & Parasuraman, R. (2011). Team Performance and Communication within Networked Supervisory Control Human-Machine Systems. *Proceedings of the Human Factors and Ergonomics Society Annual Meeting*, *55*(1), 262–266. https://doi.org/10.1177/1071181311551054

McLane, S., Turley, J. P., Esquivel, A., Engebretson, J., Smith-Akin, K. A., LoBiondo-Wood, G. L., & Zhang, J. (2010). Concept analysis of cognitive artifacts. *Advances in Nursing Science*, *33*(4), 352–361. Scopus. https://doi.org/10.1097/ANS.0b013e3181fb2ed6

McNeese, N. J., & Cooke, N. J. (2016). Team Cognition as a Mechanism for Developing Collaborative and Proactive Decision Support in Remotely Piloted Aircraft Systems. In D. D. Schmorrow & C. M. Fidopiastis (Eds), *Foundations of Augmented Cognition: Neuroergonomics and Operational Neuroscience* (Vol. 9744, pp. 198–209). Springer International Publishing. https://doi.org/10.1007/978-3-319-39952-2_20

Michailovs, S., Pond, S., Irons, J., Salmon, P. M., Visser, T. A. W., Schmitt, M., Stanton, N. A., Strickland, L., Huf, S., & Loft, S. (2025). The effect of information integration on team communication in a simulated submarine control room task. *Ergonomics*, *68*(4), 513–537. https://doi.org/10.1080/00140139.2024.2375365

Mikkelsen, C., Johansson, J., & Cooper, M. (2012). Visualization of Power System Data on Situation Overview Displays. *2012 16th International Conference on Information Visualisation*, 188–197. https://doi.org/10.1109/IV.2012.41

Nilsson, M., Laere, J. V., Susi, T., & Ziemke, T. (2012). Information fusion in practice: A distributed cognition perspective on the active role of users. *Information Fusion*, *13*(1), 60–78. Scopus. https://doi.org/10.1016/j.inffus.2011.01.005

Ozyurt, E., Doring, B., & Flemisch, F. (2013). Simulation-based development of a cognitive assistance system for Navy ships. *2013 IEEE International Multi-Disciplinary Conference on Cognitive Methods in Situation Awareness and Decision Support (CogSIMA)*, 22–29. https://doi.org/10.1109/CogSIMA.2013.6523819

Patrick, J., & Morgan, P. L. (2010). Approaches to understanding, analysing and developing situation awareness. *Theoretical Issues in Ergonomics Science*, *11*(1–2), 41–57. Scopus. https://doi.org/10.1080/14639220903009946

Petermann, F.-M., Steinke, J., Aminoff, H., Petersen, E. S., Pantelatos, L. S., & Burgén, J. (2024). Collaborative Hazard Control: Designing a Digital Fire Control Center for Enhanced Safety on Ships. *Graphics Interface*, 1–15. https://doi.org/10.1145/3670947.3671659

Power, N. (2018). Extreme teams: Toward a greater understanding of multiagency teamwork during major emergencies and disasters. *American Psychologist*, *73*(4), 478–490. APA PsycArticles (2018-23205-014). https://doi.org/10.1037/amp0000248

Power, N., & Alison, L. (2017). Redundant deliberation about negative consequences: Decision inertia in emergency responders. *Psychology, Public Policy, and Law*, *23*(2), 243–258. APA PsycArticles (2017-03597-001). https://doi.org/10.1037/law0000114

Promann, M., Wei, S., Qian, Z. C., & Chen, Y. V. (2016). The Role of Aesthetics and Perception in Raising Situation Awareness: Lessons from SpringRain. *International Journal of Human-Computer Interaction*, *32*(4), 308–324. Scopus. https://doi.org/10.1080/10447318.2016.1140951

Resick, C. J., Murase, T., Bedwell, W. L., Sanz, E., Jiménez, M., & DeChurch, L. A. (2010). Mental model metrics and team adaptability: A multi-facet multi-method examination. *Group Dynamics: Theory, Research, and Practice*, *14*(4), 332–349. APA PsycArticles (2010-25392-005). https://doi.org/10.1037/a0018822

Roberts, A. P. J., Stanton, N. A., & Fay, D. (2017). Land Ahoy! Understanding Submarine Command and Control During the Completion of Inshore Operations. *Human Factors: The Journal of the Human Factors and Ergonomics Society*, *59*(8), 1263–1288. https://doi.org/10.1177/0018720817731678

Roberts, A. P. J., Stanton, N. A., Fay, D., & Pope, K. A. (2021). It’s a circular argument: Examining how a novel configuration impacts information flow in submarine control rooms. *Applied Ergonomics*, *97*, 103534. https://doi.org/10.1016/j.apergo.2021.103534

Roberts, A. P. J., Stanton, N. A., Fay, D. T., & Pope, K. A. (2019). The effects of team co-location and reduced crewing on team communication characteristics. *Applied Ergonomics*, *81*, 102875. https://doi.org/10.1016/j.apergo.2019.102875

Saager, M., & Harre, M.-C. (2020). Modeling Distributed Situational Awareness to Improve Handling Emergency Calls in Operation Centres. In D. Harris & W.-C. Li (Eds), *Engineering Psychology and Cognitive Ergonomics. Cognition and Design* (Vol. 12187, pp. 68–78). Springer International Publishing. https://doi.org/10.1007/978-3-030-49183-3_6

Salman Nazir, Linda Johnstone Sorensen, Kjell Ivar Oevergaard, & Davide Manca. (2014). How distributed situation awareness influences process safety. *Chemical Engineering Transactions*, *36*, 409–414. https://doi.org/10.3303/CET1436069

Schelble, B. G., Flathmann, C., McNeese, N. J., O’Neill, T., Pak, R., & Namara, M. (2023). Investigating the Effects of Perceived Teammate Artificiality on Human Performance and Cognition. *International Journal of Human–Computer Interaction*, *39*(13), 2686–2701. https://doi.org/10.1080/10447318.2022.2085191

Schultze, T., Drewes, S., & Schulz-Hardt, S. (2021). A test of synergy in dynamic system control tasks. *Journal of Experimental Psychology: General*, *150*(5), 890–914. APA PsycArticles (2020-76203-001). https://doi.org/10.1037/xge0000975

Shteynberg, G., Hirsh, J. B., Bentley, R. A., & Garthoff, J. (2020). Shared worlds and shared minds: A theory of collective learning and a psychology of common knowledge. *Psychological Review*, *127*(5), 918–931. APA PsycArticles (2020-27106-001). https://doi.org/10.1037/rev0000200

Song, R., Papadimitriou, E., Negenborn, R. R., & Van Gelder, P. H. A. J. M. (2023). Distributed Situational Awareness for Maritime Autonomous Surface Ships in Mixed Waterborne Transport: An Ontology-based Framework. *2023 7th International Conference on Transportation Information and Safety (ICTIS)*, 1088–1092. https://doi.org/10.1109/ICTIS60134.2023.10243802

Stanton, N. A. (2014). Representing distributed cognition in complex systems: How a submarine returns to periscope depth. *Ergonomics*, *57*(3), 403–418. Scopus. https://doi.org/10.1080/00140139.2013.772244

Stanton, N. A., & Roberts, A. P. J. (2020). Block off: An examination of new control room configurations and reduced crew sizes examining engineered production blocking. *Cognition, Technology & Work*, *22*(1), 29–55. https://doi.org/10.1007/s10111-019-00542-x

Stanton, N. A., Roberts, A. P. J., & Fay, D. T. (2017). Up periscope: Understanding submarine command and control teamwork during a simulated return to periscope depth. *Cognition, Technology & Work*, *19*(2–3), 399–417. https://doi.org/10.1007/s10111-017-0413-7

Stanton, N. A., Roberts, A. P. J., Pope, K. A., & Fay, D. (2021). Returning to periscope depth in a circular control room configuration. *Cognition, Technology & Work*, *23*(4), 783–804. https://doi.org/10.1007/s10111-020-00654-9

Stanton, N. A., Roberts, A. P. J., Pope, K. A., & Fay, D. (2022). The quest for the ring: A case study of a new submarine control room configuration. *Ergonomics*, *65*(3), 384–406. https://doi.org/10.1080/00140139.2021.1961857

Törmä, I., Haapasaari, J., Häkkinen, J., Manngård, M., & Salokorpi, M. (2025). Enhancing Shared Situational Awareness in Remote Pilotage: A Prototype for 3D Route Briefing. *Journal of Physics: Conference Series*, *3123*(1), 012059. https://doi.org/10.1088/1742-6596/3123/1/012059

Zuo, G., Chen, J., & Dai, L. (2020). Experimental Research on Measurement of Team Situation Awareness in Nuclear Power Plants. In R. L. Boring (Ed.), *Advances in Human Error, Reliability, Resilience, and Performance* (Vol. 956, pp. 199–213). Springer International Publishing. https://doi.org/10.1007/978-3-030-20037-4_18
